# c-di-AMP Is a New Second Messenger in *Staphylococcus aureus* with a Role in Controlling Cell Size and Envelope Stress

**DOI:** 10.1371/journal.ppat.1002217

**Published:** 2011-09-01

**Authors:** Rebecca M. Corrigan, James C. Abbott, Heike Burhenne, Volkhard Kaever, Angelika Gründling

**Affiliations:** 1 Section of Microbiology, Imperial College London, London, United Kingdom; 2 Department of Life Sciences, Imperial College London, London, United Kingdom; 3 Institute of Pharmacology, Hannover Medical School, Hannover, Germany; Dartmouth Medical School, United States of America

## Abstract

The cell wall is a vital and multi-functional part of bacterial cells. For *Staphylococcus aureus,* an important human bacterial pathogen, surface proteins and cell wall polymers are essential for adhesion, colonization and during the infection process. One such cell wall polymer, lipoteichoic acid (LTA), is crucial for normal bacterial growth and cell division. Upon depletion of this polymer bacteria increase in size and a misplacement of division septa and eventual cell lysis is observed. In this work, we describe the isolation and characterization of LTA-deficient *S. aureus* suppressor strains that regained the ability to grow almost normally in the absence of this cell wall polymer. Using a whole genome sequencing approach, compensatory mutations were identified and revealed that mutations within one gene, *gdpP* (GGDEF domain protein containing phosphodiesterase), allow both laboratory and clinical isolates of *S. aureus* to grow without LTA. It was determined that GdpP has phosphodiesterase activity *in vitro* and uses the cyclic dinucleotide c-di-AMP as a substrate. Furthermore, we show for the first time that c-di-AMP is produced in *S. aureus* presumably by the *S. aureus* DacA protein, which has diadenylate cyclase activity. We also demonstrate that GdpP functions *in vivo* as a c-di-AMP-specific phosphodiesterase, as intracellular c-di-AMP levels increase drastically in *gdpP* deletion strains and in an LTA-deficient suppressor strain. An increased amount of cross-linked peptidoglycan was observed in the *gdpP* mutant strain, a cell wall alteration that could help bacteria compensate for the lack of LTA. Lastly, microscopic analysis of wild-type and *gdpP* mutant strains revealed a 13–22% reduction in the cell size of bacteria with increased c-di-AMP levels. Taken together, these data suggest a function for this novel secondary messenger in controlling cell size of *S. aureus* and in helping bacteria to cope with extreme membrane and cell wall stress.

## Author Summary

*Staphylococcus aureus* is an important human pathogen that colonizes the nares and skin of both sick and healthy individuals and causes a variety of infections ranging from superficial skin to invasive infections. The ability of this bacterium to cause disease depends on many factors and is, in part, due to multi-functional cell surface structures. One such structure is lipoteichoic acid (LTA), which is crucial for bacterial growth. In this study we show that LTA is also important for growth of a clinically relevant community-acquired methicillin resistant *S. aureus* (CA-MRSA) strain and not only for laboratory strains as previously described. We set out to investigate if *S. aureus* can find a way to survive without LTA and identified strains that can grow and divide almost normally in its absence. Using a whole genome sequencing approach, we found that alterations in one gene, *gdpP*, allow these strains to grow in the absence of LTA. We show that this mutation causes an increase in the recently identified signaling molecule, c-di-AMP, within the cell. Therefore, with this study we provide information on one of the first functions of this novel secondary messenger, which is in helping bacteria to cope with extreme cell wall stress.

## Introduction

*Staphylococcus aureus* is a very prevalent human pathogen that permanently colonizes the nares and skin of approximately 20% of the population, while another 60% are colonized transiently [1]. Infections caused by this pathogen are becoming increasingly more difficult to treat due to its resistance to antibiotic therapy. Where once methicillin was the antibiotic of choice, now only around 60% of *S. aureus* isolates remain sensitive to this drug. There has also been a rise in the number of community acquired methicillin resistant *S. aureus* (CA-MRSA) cases in recent years often resulting in severe skin and soft tissue infections as well as invasive diseases such as sepsis, necrotizing pneumonia or osteomyelitis [2], [3]. The ability of *S. aureus* to cause such a wide range of diseases depends on many factors and is, in part, due to the diverse functions that are linked to its cell envelope. A myriad of proteins are embedded in this structure that allow bacteria to take up nutrients and adhere to diverse surfaces or niches within the human host. It also protects bacteria from environmental insults and at the same time allows the cells to sense and respond to changes in their surroundings, a function crucial for the survival of this pathogen in the host. In addition, the cell wall helps bacteria to maintain their shape and functions to counteract the high internal turgor pressure. Because the cell envelope has such essential functions, it also forms a weak point of the cell, as the inhibition of enzymes required for its synthesis is often lethal or leads to virulence defects. Therefore, this structure has been, and remains, an attractive target for therapeutic interventions.

A typical cell wall of Gram-positive bacteria consists of proteins, peptidoglycan (PG) and the cell wall polymers wall teichoic acid (WTA), which is covalently linked to PG, and lipoteichoic acid (LTA), a polymer anchored to the outside of the bacterial membrane via a lipid moiety [4], [5], [6]. Synthesis of these cell wall components is highly coordinated and any mistakes can lead to cell lysis and death. From studies on the Gram-positive model organism *Bacillus subtilis,* it has emerged that PG and WTA synthesis enzymes form multi-protein complexes, which are further linked in this organism with cytoplasmic cell shape determining proteins, thereby coordinating and physically linking extracellular and intracellular synthesis processes [7], [8], [9], [10].

*S. aureus* LTA is an anionic cell wall polymer consisting of a linear chain of glycerolphosphate repeating units that is anchored via a glycolipid to the membrane [11]. The glycerolphosphate subunits are derived from the head group of the membrane lipid phosphatidylglycerol and polymerized on the outside of the cell by the membrane-linked lipoteichoic synthase enzyme LtaS to form the backbone chain [12], [13], [14], [15]. A large fraction of the glycerolphosphate subunits are substituted with D-alanine residues, a modification known to play a key role in the resistance of *S. aureus* and several other Gram-positive pathogens to cationic antimicrobial peptides [16], [17], [18], [19]. A function for this polymer in the formation of biofilms has been identified and multiple interactions between LTA and eukaryotic cells have been described [20], [21]. An interaction between LTA and macrophage scavenger receptors is thought to occur and help the host to clear bacterial infections [22], [23]. In agreement with this suggestion, scavenger receptor knockout mice are more susceptible to infection with *S. aureus* [24]. This polymer has also been shown to act as a ligand for Draper, a phagocytic receptor in *Drosophila*, which upon binding of LTA results in the phagocytosis of *S. aureus* by *Drosophila* hemocytes [25]. On the other hand, LTA is also thought to act as an anti-inflammatory molecule on skin cells by suppressing the TLR-3-mediated responses upon skin injury, a key pathway in the induction of inflammation [26].

Recently defined mutants lacking the entire LTA polymer have been constructed and phenotypic analysis indicated an important role for this polymer for normal bacterial growth and morphology [13], [27], [28], [29], [30]. LTA-deficient *S. aureus* strains have severely impaired growth and can initially only be propagated in medium containing high salt or sucrose concentrations, which are thought to act as osmoprotectants, or at low temperature [13], [27]. Even under conditions permissive for growth, these cells have severe morphological defects, such as an increased cell size and the tendency to clump. In addition, misplacement of cell division septa is observed, highlighting that the lack of LTA on the outside of the cell negatively affects fundamental processes in the cytoplasm of the cell. However, it is currently not known how these processes are coordinated.

In this study we set out to investigate if *S. aureus* can find a way to survive without LTA and identified LTA-deficient suppressor strains that can grow and divide almost normally in the absence of this multi-functional cell wall polymer. Using a whole genome sequencing approach, it was determined that these strains have acquired mutations in a gene encoding a protein named GdpP (for GGDEF domain protein containing phosphodiesterase). We show that as a consequence of this mutation, the intracellular levels of the novel cyclic dinucleotide c-di-AMP increase drastically in the suppressor strain and *gdpP* mutant laboratory or CA-MRSA strains. This provides the first experimental evidence that c-di-AMP is produced in *S. aureus* and that GdpP functions as a c-di-AMP phosphodiesterase *in vivo.* With this study we provide information on one of the first functions of this novel secondary messenger, which is in helping bacteria to cope with extreme cell wall stress in addition to controlling the cell size of *S. aureus,* as revealed by microscopic analysis.

## Results

### Construction of an LTA negative *S. aureus* RN4220 strain

**Figure 1:**
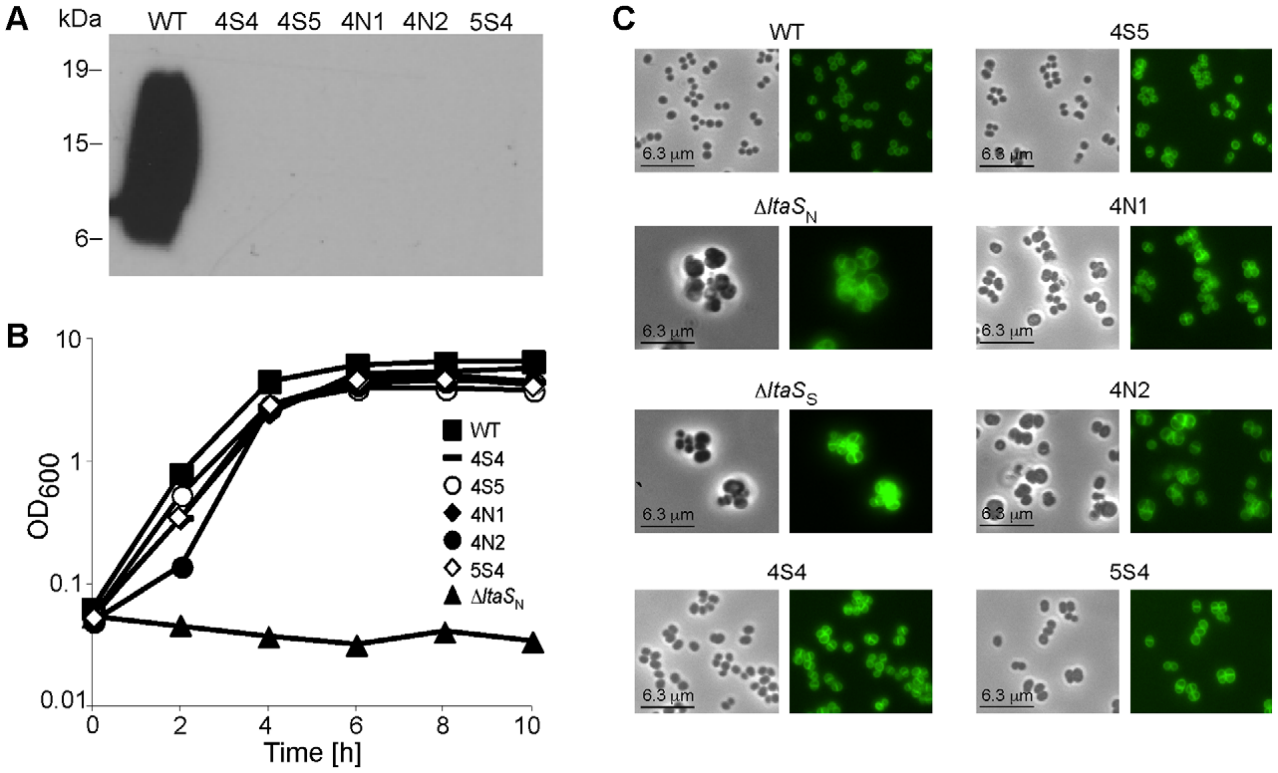
Characterization of Δ*ltaS* suppressor strains. (A) LTA detection by western blot. Cell-associated LTA isolated from strain SEJ1 (WT) and the five suppressor strains (4S4, 4S5, 4N1, 4N2 and 5S4) was analyzed by western blot. The positions of protein molecular mass markers (in kDa) are indicated on the left. (B) Bacterial growth curves. Overnight cultures of SEJ1 (WT) and the five suppressor strains (4S4, 4S5, 4N1, 4N2 and 5S4) were grown in TSB. Strain SEJ1Δ*ltaS*_N_ (Δ*ltaS*_N_) was grown overnight in TSB 7.5% NaCl. All cultures were washed 3 times in TSB and diluted to a starting OD_600_ of 0.05 in TSB. Growth was monitored over a 10 h period. (C) Microscopic analysis of WT and LTA-negative strains. SEJ1 (WT) and the LTA-negative strains (4S4, 4S5, 4N1, 4N2 and 5S4) were grown in TSB to log-phase and strains SEJ1Δ*ltaS*_N_ (Δ*ltaS*_N_) and SEJ1Δ*ltaS*_S_ (Δ*ltaS*_S_) were grown in TSB containing 7.5% NaCl or 40% sucrose, respectively. Samples were prepared for microscopy analysis and bacteria stained with BODIPY-vancomycin as described in the materials and methods section.

In order to determine whether it is possible for *S. aureus* to compensate for the cell wall stresses introduced by deleting LTA, the RN4220-derived *ltaS* deletion strains SEJ1Δ*ltaS*_N_ and SEJ1Δ*ltaS*_S_ were created and the lack of LTA confirmed by western blot (Figure S1A in Text S1, data not shown). These strains were constructed under high osmotic conditions in medium containing 7.5% NaCl (N) or 40% sucrose (S) that are, as previously shown, permissive for growth of *S. aureus* in the absence of LTA (Figure S1B in Text S1) [27]. However, in contrast to this previous study [27], growth of our *ltaS* deletion strains were dependent on osmoprotectants at both 30°C and 37°C. Plating efficiencies for the Δ*ltaS*_N_ strain decreased from 3.5×10^8^ CFU/ml on 7.5% NaCl containing TSA plates to 8×10^1^ CFU/ml on TSA plates at 37°C and similar low CFUs were obtained at 30°C. SEJ1Δ*ltaS* bacteria had aberrant cell morphologies even under conditions permissive for growth and displayed an enlarged cell size, a tendency to cluster and a misplacement of division sites (Figure 1C) [13], [27]. In summary, our LTA-negative *S. aureus* RN4220 strains are viable under osmotically stabilizing conditions but not in TSB medium and display the expected morphological and cell division defects.

### Characterization of *S. aureus* suppressor strains that can grow without LTA

When SEJ1Δ*ltaS* strains were plated on TSA plates without the addition of sucrose or salt a small number of colonies were obtained. We hypothesized that these colonies arose from bacteria that had acquired compensatory mutation(s) that allow bacteria to grow in the absence of LTA. To analyze this further, five independently isolated suppressor colonies were passed four times in TSB to improve growth. As expected, LTA was still absent in the five suppressor strains 4S4, 4S5, 4N1, 4N2 and 5S4 (Figure 1A), however growth of these strains now more closely resembled that of the parental strain SEJ1 (Figure 1B). Examination of the five suppressor strains by phase contrast and fluorescence microscopy revealed a near WT cell size and a considerable improvement in the accuracy of division site placement, although misplacement of septa still occurred in some cells (Figure 1C).

To further investigate the cell wall properties of these LTA-negative suppressor strains additional assays were performed. The suppressor strains were two to four-fold more susceptible to lysostaphin, nisin, vancomycin, oxacillin, penicillin G and daptomycin (Table S1 in Text S1). In addition, the suppressor strains lysed faster than the control strain in autolysis assays (Figure S2A in Text S1) but had slightly reduced amounts of cell wall-associated hydrolytic enzymes as determined by zymogram assays (Figure S2B in Text S1). LTA production was restored in two suppressor strains, 4S5 and 5S4, by introducing the complementation vector pCN34-*ltaS*. The amount of cell-associated hydrolytic enzymes increased in these strains (Figure S2C in Text S1), suggesting that LTA has a role in regulating autolysin levels. This is supported by the observations of Oku *et al* [27] who noted an even greater reduction in the amount of autolysins for their *ltaS* mutant strain, which could again be complemented. Taken together, *S. aureus* suppressor strains that can grow in the absence of LTA can be isolated readily and the morphology and cell division pattern defects are significantly improved in these strains. However, differences in autolysis and susceptibility to cell wall active antibiotics are indicative of remaining changes in the cell wall properties of these strains.

### Identification of mutations in LTA-negative *S. aureus* suppressor strains

It seems likely that the LTA-negative *S. aureus* suppressor strains 4S4, 4S5, 4N1, 4N2 and 5S4 have acquired mutations elsewhere on the chromosome that allow for improved cell division and growth. A whole genome sequencing approach was used to identify such sequence alterations (for details see materials and methods section). In total, ten genes within these five strains contained mutations with high confidence scores. Five of these mutations were discarded as they were also present in strain SEJ1Δ*ltaS* pCN34-*ltaS,* an intermediate strain used for the construction of the *ltaS* deletion strains that still contains an intact copy of the *ltaS* gene. It is likely that these mutations were introduced during the temperature shift necessary to create the *ltaS* deletion. The mutations in the remaining five genes were at different positions and consisted of nonsense mutations, amino acid substitutions or DNA inversions (Table 1). The five genes included SAOUHSC_00015, a conserved hypothetical protein with putative diguanylate cyclase and phosphoesterase activity and the only gene mutated in all five suppressor strains; SAOUHSC_01104, encoding for the succinate dehydrogenase SdhA, a TCA cycle enzyme; SAOUHSC_01358, which encodes for a putative permease; SAOUHSC_02001, a conserved hypothetical protein with weak homology to a fusaric acid transporter, and SAOUHSC_02407, a conserved hypothetical protein with homology to DisA, a DNA integrity scanning protein from *B. subtilis*. In each suppressor strain two to four of these five genes were mutated and all mutations were confirmed by re-sequencing all five genes in each suppressor strain.

**Table 1:** Mutations present in SEJ1Δ*ltaS* suppressor strains.

	*S. aureus* NCTC8325 gene number				
Strain	SAOUHSC_00015	SAOUHSC_01104	SAOUHSC_01358	SAOUHSC_02001	SAOUHSC_02407
**4S4**	Inverted 18,924–19,002 (AA203–227 flipped)	C 1,065,691 G (Thr119Arg)	C 1,300,654 G (Pro287Arg)	WT	C 2,235,383 A (Leu193Phe)
**4S5**	G 19,114 A (Gly266Asp)	WT	G 1,300,870 A (Gly359Asp)	WT	WT
**4N1**	A insertion 19,209 (Asn300stop)	WT	A 1,299,810 T (Lys6stop)	WT	G 2,235,253A (Thr236Ile)
**4N2**	Inverted 19,477–19,758 (Ile416stop)	WT	WT	A 1,911,510 T (Arg207stop)	WT
**5S4**	Deleted T 19,205 (Val319stop)	WT	C 1,300,642 T (Pro283Leu)	C 1,911,723 T (Gln278stop)	WT

Numbers indicate site of base substitutions in the respective SEJ1Δ*ltaS* suppressor strain as compared to the SEJ1Δ*ltaS* reference strain sequence with numbers given based on the *S. aureus* NCTC8325 genome sequence. Resulting amino acid changes in corresponding proteins are shown in parenthesis. WT indicates that the sequences were identical between SEJ1Δ*ltaS* reference strain and suppressor strain.

As mentioned above, the suppressor strains were passed four times in broth culture to improve growth before phenotypic and sequence analysis. Therefore, one or more of the mutations listed in Table 1 may have arisen during the passing steps to aid with growth and not as a direct consequence of assisting growth of the LTA-negative strains. To address this, chromosomal DNA was isolated from the original suppressor colonies of strains 4S4, 4S5, 4N1, 4N2 and 5S4 and the five genes in question were sequenced. Only the mutations in gene SAOUHSC_00015 were present in all five strains. Thus, it appears that inactivation of SAOUHSC_00015, already named in some *S. aureus* strains GdpP (for GGDEF domain protein containing phosphodiesterase), compensates for the lack of LTA. The remaining mutations in the four other genes are possibly accessory and function to improve growth.

### Disruption of *gdpP* allows for the growth of an LTA-deficient strain of *S. aureus*

**Figure 2:**
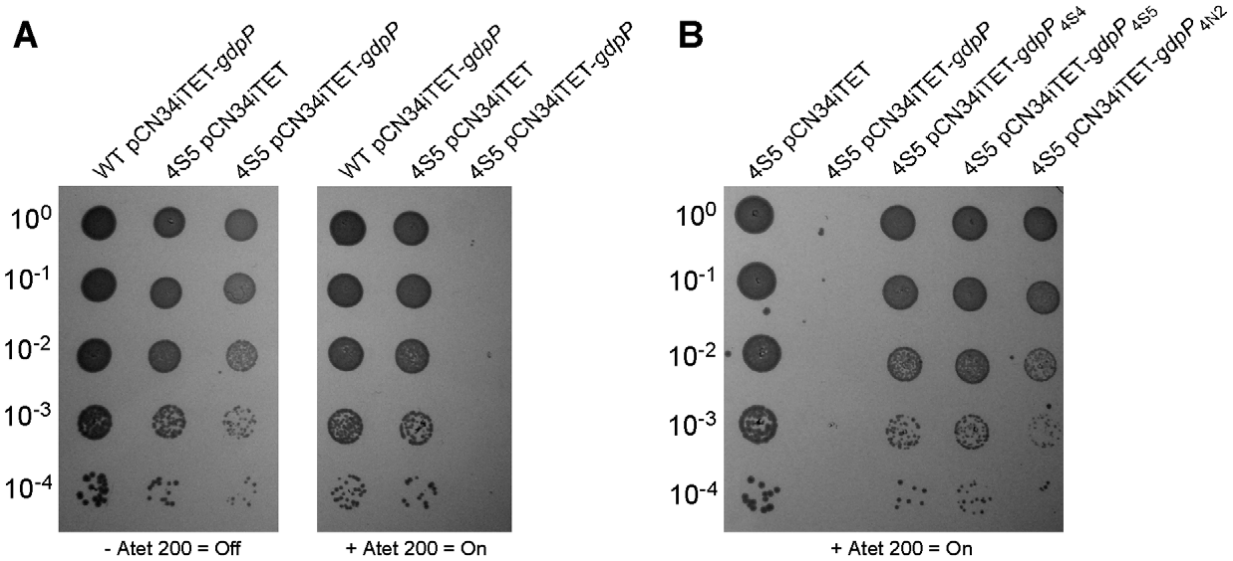
Expression of different *gdpP* alleles in an LTA-negative suppressor strain and their effect on growth. (A) Serial dilutions (indicated on the left) of WT SEJ1 containing the Atet-inducible GdpP expression vector (WT pCN34iTET*-gdpP*), the suppressor strain 4S5 containing the empty vector (4S5 pCN34iTET) or the GdpP expression vector (4S5 pCN34iTET-*gdpP*) were spotted on TSA plates containing either no (left panel) or 200 ng/ml Atet (right panel). (B) The mutated *gdpP* alleles from suppressor strains 4S4, 4S5 and 4N2 (indicated above panel) were expressed from pCN34iTET in the strain 4S5. Serial dilutions of were spotted on TSA 200 ng/ml Atet plates to induce protein expression from the plasmid vector.

**Figure 3:**
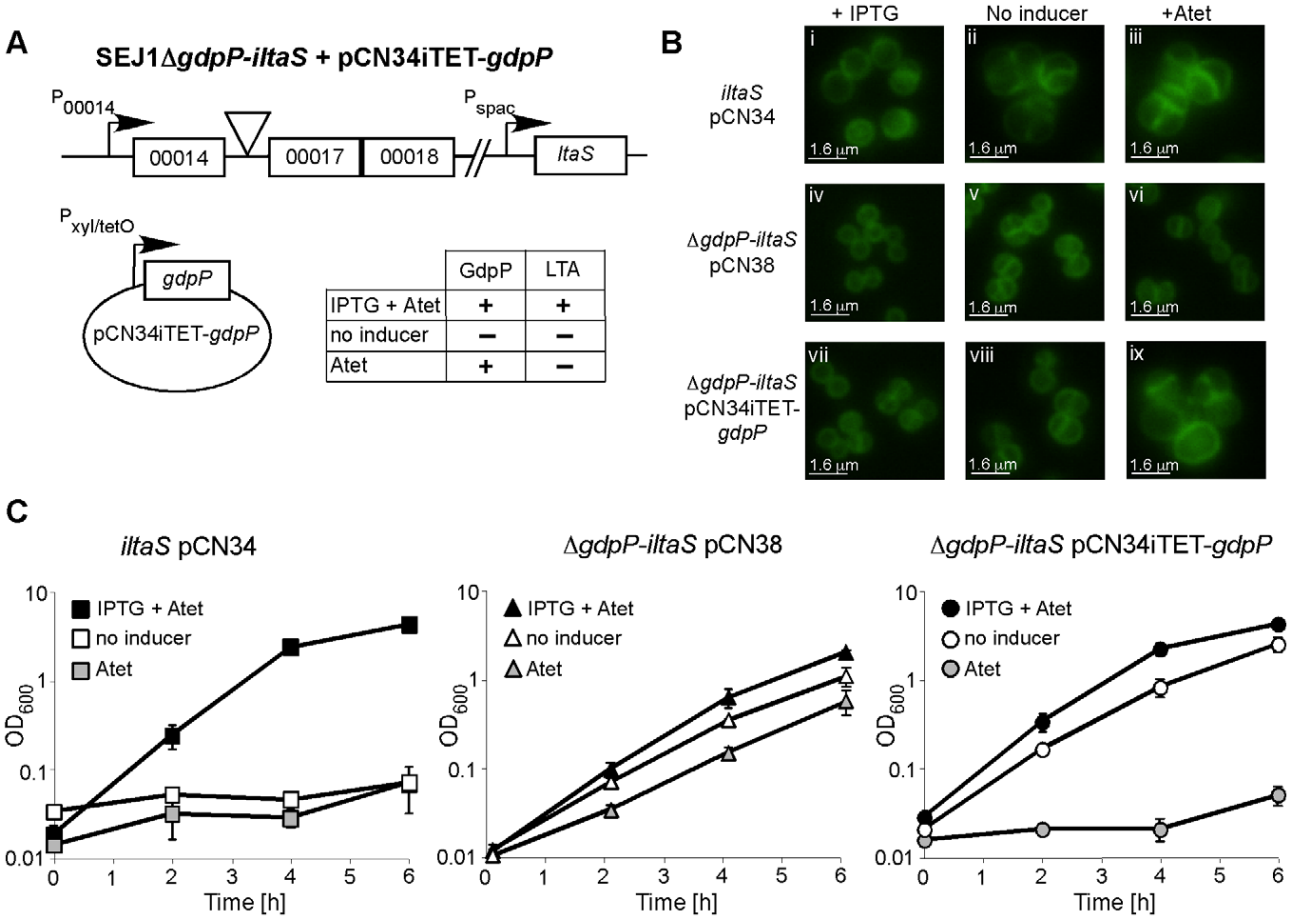
Deletion of *gdpP* can rescue the dependence of RN4220*iltaS* on IPTG. (A) Schematic representation of *S. aureus* strain SEJ1Δ*gdpP*-*iltaS* pCN34iTET-*gdpP*, containing an inframe *gdpP* deletion, the *ltaS* gene under IPTG inducible P_spac_ control and the complementing vector pCN34iTET-*gdpP*. The effects of the addition of the inducers on the production of LTA and GdpP are indicated. (B) Microscopic analysis of strains RN4220*iltaS* pCN34 (*iltaS* pCN34; i–iii), SEJ1Δ*gdpP*-*iltaS* pCN38 (Δ*gdpP*-*iltaS* pCN38; iv–vi) and SEJ1Δ*gdpP*-*iltaS* pCN34iTET-*gdpP* (Δ*gdpP*-*iltaS* pCN34iTET-*gdpP*; vii–ix). Strains were grown overnight in the presence of IPTG, washed and grown for 8 h in TSB containing 1mM IPTG (left panel), TSB (no inducer – middle panel) or TSB containing 100 ng/ml Atet (right panel). Bacteria were stained with BODIPY-vancomycin and prepared for microscopy analysis as described in the materials and methods section. Larger fields of view are shown in Figure S3B in Text S1 (C) Bacterial growth in the presence or absence of inducer. Strains RN4220*iltaS* pCN34 (*iltaS* pCN34), SEJ1Δ*gdpP*-*iltaS* pCN38 (Δ*gdpP*-*iltaS* pCN38) and SEJ1Δ*gdpP*-*iltaS* pCN34iTET-*gdpP* (Δ*gdpP*-*iltaS* pCN34iTET-*gdpP*) were grown overnight in the presence of IPTG, washed and diluted in either TSB containing 1mM IPTG +100 ng/ml Atet (black symbols), TSB containing 100 ng/ml Atet (grey symbols) or just plain TSB (no inducer – white symbols). After 4 h cultures were diluted 1:100 into fresh medium to maintain them in the exponential growth phase and this OD_600_ is plotted as T = 0. Growth was then continued for a further 6 h. Growth curves were performed in triplicate and the average and standard deviations were plotted.

If disruption of *gdpP* compensates for a lack of LTA, one would predict that introducing a WT copy of *gdpP* into a suppressor strain should be lethal, while the introduction of any of the mutant *gdpP* alleles present in the suppressor strains should not prevent growth. This was indeed the case, and the expression of WT *gdpP* from an anhydrotetracycline (Atet) inducible promoter containing plasmid but not the expression of the mutant *gdpP* alleles obtained from suppressor strains 4S4 (inverted sequence, inframe), 4S5 (point mutation) or 4N2 (stop codon) prevented growth of strain 4S5 (Figure 2B). As controls, introduction of the empty vector pCN34iTET or uninduced pCN34iTET-*gdpP* into the suppressor strain 4S5 had no effect on bacterial growth (Figure 2A, left panel) and expression of GdpP in WT SEJ1 also had no effect on growth, demonstrating that expression of GdpP is not toxic per se (Figure 2A, right panel). These results provide further evidence that disruption of *gdpP* is essential for the survival of LTA-negative *S. aureus* suppressor strains. However, the suppressor strains contain other known mutations (see text and Table 1), therefore we set out to recreate the system in an *S. aureus* strain without these additional mutations. To this end, the *S. aureus* strain SEJ1Δ*gdpP-iltaS* with a silent *gdpP* deletion and IPTG inducible *ltaS* expression was created (Figure 3A). Normally, the growth of an inducible *ltaS* strain is dependent on IPTG [13], however this should no longer be the case upon deletion of *gdpP* and expression of GdpP from a plasmid should restore IPTG-dependent growth to this strain. *S. aureus* strains SEJ1*-iltaS* pCN34 (*iltaS* pCN34) and SEJ1Δ*gdpP-iltaS* containing the GdpP expression plasmid (Δ*gdpP iltaS* pCN34iTET-*gdpP*) were used to test this experimentally. All strains grew in the presence of IPTG (Figure 3C, black filled symbols), produced LTA (Figure S3A in Text S1) and displayed a normal cell shape and placement of division septa (Figure 3B and S3B in Text S1, panels i, iv & vii). It is interesting to note that SEJ1Δ*gdpP-iltaS* bacteria, which contain a deletion of the *gdpP* gene, appear to have a reduced cell size (Figure 3B, iv & vii) and this will be analyzed in more detail later on. In the absence of IPTG, LTA was no longer produced (Figure S3A in Text S1) and as expected growth of the inducible *ltaS* strain ceased (Figure 3C, *iltaS* pCN34 – white and grey filled squares). Morphologically the cells appeared enlarged, clumped and showed aberrant cell division (Figure 3B and S3B in Text S1, panels ii & iii). However, as predicted growth of the inducible *ltaS* strain with the *gdpP* deletion continued even in the absence of IPTG (Figure 3C, Δ*gdpP-iltaS* pCN38 – white and grey filled triangles) and the morphology of the cells was much improved showing more regular cell sizes and division (Figure 3B and S3B in Text S1, panels v, vi & viii). Addition of Atet (grey symbols) for the expression of GdpP from the complementation vector restored the IPTG-dependent growth phenotype and strain SEJ1Δ*gdpP-iltaS* pCN34iTET-*gdpP* ceased to grow in the absence of IPTG (Figure 3C, Δ*gdpP iltaS* pCN34iTET-*gdpP* - grey circles) and cells displayed an aberrant morphology (Figure 3B and S3B in Text S1, panel ix). In summary, it can now be concluded that deleting the *gdpP* gene allows for the growth of an LTA-deficient strain of *S. aureus*.

### LTA is important for growth of a clinical isolate of *S. aureus* and disruption of *gdpP* can compensate for the growth inhibition

**Figure 4:**
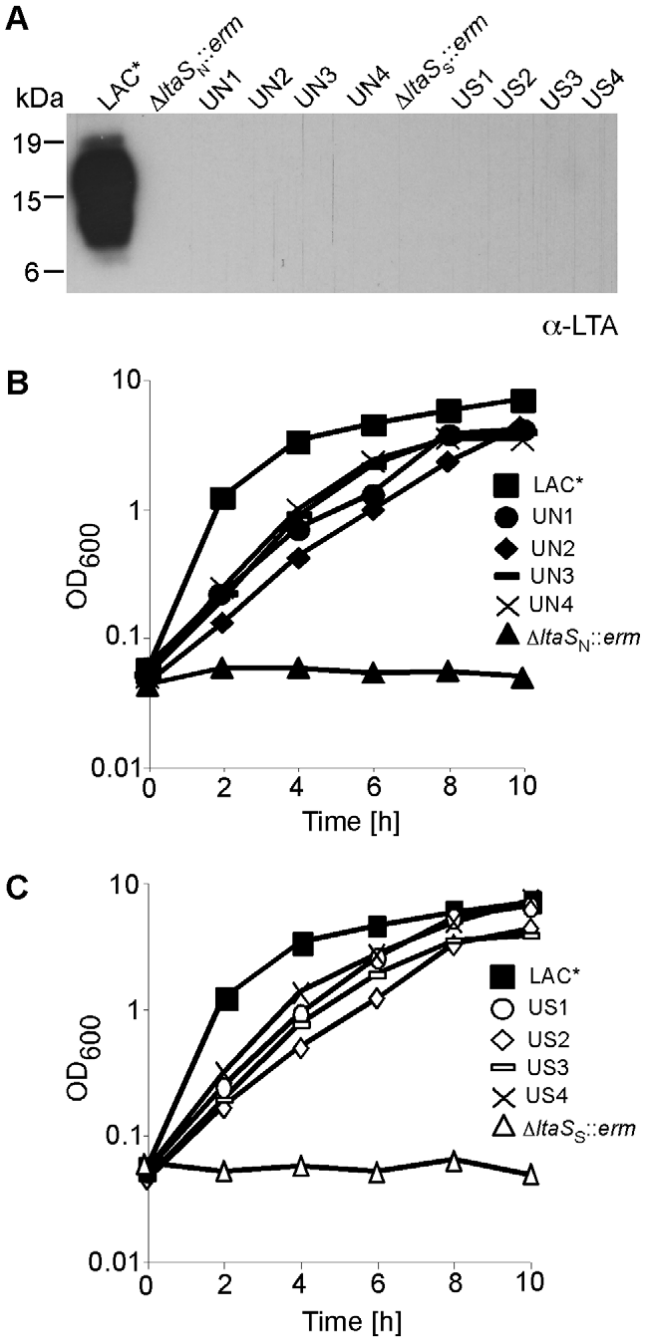
Growth and LTA production of CA-MRSA LAC* and LAC*Δ*ltaS* strains. (A) LTA detection by western blot. *S. aureus* LAC* (WT) and the eight suppressor strains UN1, UN2, UN3, UN4, US1, US2, US3 and US4 were grown in TSB. LAC*Δ*ltaS*_N_ *::erm* was grown in TSB containing 7.5% NaCl and LAC*Δ*ltaS*_S_ *::erm* in TSB containing 40% sucrose and LTA detected by western blot. (B) and (C) Bacterial growth curves. Overnight cultures of strains described above were washed 3 times and diluted to a starting OD_600_ of 0.05 in TSB and OD_600_ readings determined every 2 h for 10 h.

The experiments described above confirm that deletion of *gdpP* compensates for the lack of LTA in an RN4220-derived *S. aureus* strain. However, this strain is a chemically mutagenized laboratory strain that contains known mutations and defects in regulatory systems [31], [32], [33], [34]. To determine if LTA is also important for growth of other *S. aureus* isolates, and whether mutations in *gdpP* can compensate for the lack of LTA, the *ltaS* gene was deleted in the erythromycin sensitive community-acquired MRSA (CA-MRSA) strain LAC* [35]. Strains LAC*Δ*ltaS*_N_::*erm* and LAC*Δ*ltaS*_S_::*erm* with complete *ltaS* deletions could be obtained on TSA plates containing 7.5% NaCl or 40% sucrose and the absence of LTA was confirmed by western blot (Figure 4A). Both LAC*Δ*ltaS*::*erm* strains could initially only grow in the presence but not in the absence of osmoprotectants (Figure 4B). Identical to the RN4220 *ltaS*-deletion strains, LAC*Δ*ltaS*::*erm* suppressor colonies could be obtained on TSA plates. These strains did not produce LTA but were now able to grow in the absence of osmoprotectants (Figure 4). To establish whether the LAC*Δ*ltaS*::*erm* suppressor strains had acquired mutations within *gdpP*, this gene was sequenced from eight independently isolated suppressor strains. Of these eight strains, five had mutations in the *gdpP* gene (Table S2 in Text S1). These results show that LTA is also important for the growth of a CA-MRSA strain and indicate that, as observed in RN4220, inactivation of *gdpP* provides a mechanism that allows LAC* to grow in the absence of LTA.

### *S. aureus* GdpP has *in vitro* c-di-AMP phosphodiesterase activity

**Figure 5:**
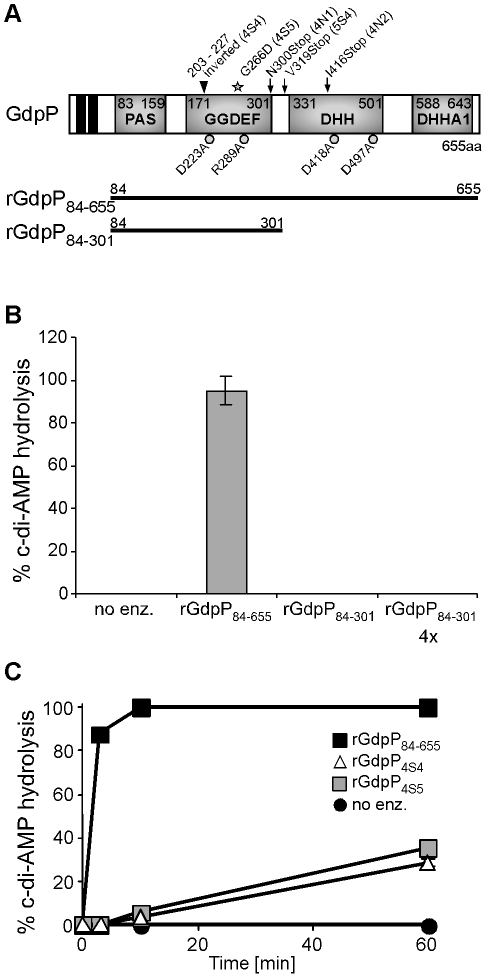
*In vitro* phosphodiesterase activity of WT and mutant GdpP variants. (A) Schematic representation of the GdpP protein. The N-terminus of the protein contains 2 transmembrane helices (black boxes), a degenerate PAS domain (residues 83–159), a GGDEF domain (residues 171–301), a DHH domain (residues 331–501) and a DHHA1 domain (residues 588–643). Protein domains expressed as recombinant proteins are indicated by the thick black lines. Amino acids mutated to alanines by site-directed mutagenesis are indicated by grey circles and residues altered in suppressor strains are indicated by a grey star (amino acid substitution – 4S5), by black arrows (stop codons – 4N1, 4N2 and 5S4) or by a triangle (inframe amino acid inversion – 4S4). (B) and (C) Phosphodiesterase activity of rGdpP proteins. (B) Enzyme reactions were set up in assay buffer containing 20 µM c-di-AMP and either no enzyme (no enz.), 1 µM rGdpP_84–655_, 1 µM rGdpP_84–301_ or 4 µM rGdpP_84–301_. Reactions were stopped after 60 min and% c-di-AMP hydrolysis calculated based on nucleotide peak areas following HPLC separation. (C) Enzyme reactions were set up as described above using the recombinant GdpP variants indicated in the legend. Reactions were stopped after 3, 10, or 60 min and% c-di-AMP determined as described above. Four independent experiments were performed and the average value and standard deviation plotted.

*S. aureus* GdpP contains two N-terminal transmembrane helices followed by a degenerated PAS sensory domain (Pfam00989), a GGDEF domain (Pfam00990), a DHH domain (Pfam01368) and a DHH-associated DHHA1 domain (Pfam02272) (Figure 5A). GGDEF domains are usually associated with proteins containing c-di-GMP cyclase or phosphodiesterase activity [36], [37] and DHH/DHHA1 domain-containing proteins often function as phosphatases or phosphoesterases [38]. The *B. subtilis* protein YybT is a close homologue to *S. aureus* GdpP and recently it was shown that recombinant *B. subtilis* YybT has strong phosphodiesterase activity contained within the DHH/DHHA1 domains [39]. It was further suggested that the cyclic dinucleotide c-di-AMP is the physiological substrate and is converted to 5′-pApA by YybT [39]. In addition, it was found that the GGDEF domain of YybT has weak ATPase activity but no c-di-GMP or c-di-AMP cyclase activity [39]. To investigate whether *S. aureus* GdpP, like *B. subtilis* YybT, is a c-di-AMP phosphodiesterase an N-terminally His-tagged fragment of GdpP spanning amino acids 84–655 and containing the PAS, GGDEF and DHH/DHHA1 domains was expressed and purified from *E. coli* extracts (Figures 5A and S4 in Text S1). Incubation of c-di-AMP with the recombinant rGdpP_84–655_ protein resulted in the complete conversion of c-di-AMP to 5′-pApA. This was initially determined by mass spectrometry analysis (data not shown) and subsequently quantified by separating reaction products by HPLC and integrating the nucleotide peak areas using c-di-AMP and 5′-pApA standards as controls (Figure 5B). Recombinant rGdpP_84-301_ protein containing only the PAS and GGDEF domains was also used in this assay and even when present in 4-fold higher amounts did not show any phosphodiesterase activity (Figure 5B). Therefore, identical to *B. subtilis* YybT, recombinant *S. aureus* GdpP has *in vitro* c-di-AMP phosphodiesterase activity and the DHH/DHHA1 domain is essential for this activity. Purified *S. aureus* rGdpP_84–655_ did not show any, or at the most very weak, ATPase activity *in vitro* (data not shown). However reaction conditions were not further optimized, as the phosphodiesterase activity appears to be the biologically relevant activity (see below).

### Disruption of the c-di-AMP phosphodiesterase activity of GdpP is essential for the survival of LTA-negative *S. aureus* strains

The *gdpP* mutations present in three of the five sequenced RN4220 LTA suppressors (4N1, 4N2 and 5S4) lead to stop codons in or before the DHH/DHHA1 domain, which will automatically disrupt the phosphodiesterase activity (Figure 5A). On the other hand, the *gdpP* mutations in 4S4 and 4S5 lead to the expression of GdpP variants with a 24 amino acid inversion (but still in frame) and a G266D amino acid substitution. To test if these alterations affect phosphodiesterase activity, rGdpP_4S4_ and rGdpP_4S5_ variants (comprising amino acids 84–655) were produced (Figure S4 in Text S1) and the phosphodiesterase activity measured. While all of the input c-di-AMP was hydrolyzed by WT rGdpP_84–655_ in less than ten minutes, recombinant rGdpP_4S4_ and rGdpP_4S5_ variants converted less than 40% of the input substrate to 5′-pApA in one hour (Figure 5C). These results provide evidence that a disruption of the phosphodiesterase function of GdpP is responsible for compensating for a lack of LTA in suppressor strains.

**Figure 6:**
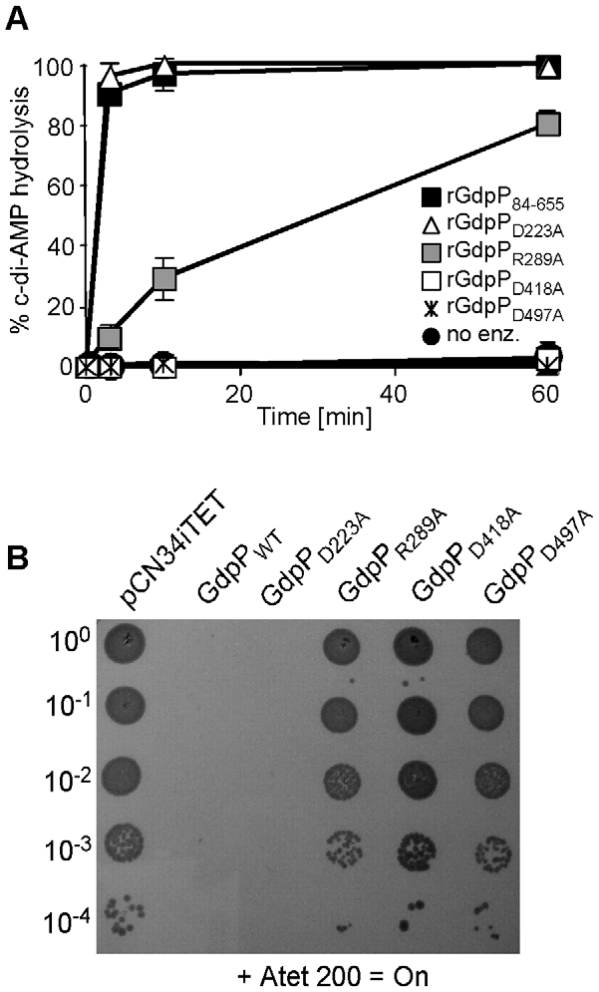
Disruption of GdpP phosphodiesterase activity compensates for a lack of LTA. (A) Phosphodiesterase activity of different rGdpP variants. Enzyme reactions were set up in assay buffer containing 20 µM c-di-AMP and 1 µM of the recombinant rGdpP variants indicated in the legend. Reactions were stopped after 3, 10, or 60 min and% c-di-AMP determined based on nucleotide peak areas following HPLC separation. Four independent experiments were performed and the average value and standard deviation plotted. (B) Bacterial growth assay. Alanine substituted GdpP variants were expressed from pCN34iTET in the suppressor strain 4S5. Serial dilutions of 4S5 containing the plasmid indicated above the panel were spotted on TSA plates containing 200 ng/ml Atet.

In *B. subtilis* YybT, amino acids D225 and R291 in the GGDEF domain and residues D420 and D499 in the DHH/DHHA1 were identified as key residues for the weak ATPase activity and the phosphodiesterase activity, respectively [39]. Alanine substitutions at the corresponding positions D223, R289, D418 and D497 in *S. aureus* GdpP (Figure 5A) were made and recombinant proteins expressed and purified (Figure S4 in Text S1) for use in *in vitro* phosphodiesterase assays or were expressed from the Atet-inducible vector pCN34iTET in *S. aureus* strain 4S5 in order to investigate which activity needs to be inactivated to allow for the survival of the LTA-negative suppressor strain. As expected, recombinant rGdpP_D418A_ and rGdpP_D497A_ with substitutions of key residues in the DHH/DHHA1 domains lacked phosphodiesterase activity (Figure 6A). Interestingly, the rGdpP_R289A_ variant with a substitution in the GGDEF domain also had reduced *in vitro* phosphodiesterase activity, while the activity of the rGdpP_D223A_ enzyme was identical to WT rGdpP_84–655_ (Figure 6A). The survival of the LTA-negative suppressor correlated with the defect in phosphodiesterase activity as expression of GdpP_R289A_, GdpP_D418A_ or GdpP_D497A_ did not affect the growth of strain 4S5, while expression of GdpP_D223A_ with WT phosphodiesterase activity prevented the growth of this strain (Figure 6B). Therefore, the ability of *S. aureus* to grow in the absence of LTA seems to be independent of any potential ATPase activity GdpP may have, but the survival depends on the disruption of the phosphodiesterase activity and possibly a concurrent increase in c-di-AMP concentration in the cell.

### The *S. aureus* enzyme DacA is a c-di-AMP cyclase

Our results thus far have shown that GdpP has *in vitro* c-di-AMP phosphodiesterase activity. For this activity to be of any biological significance, *S. aureus* needs to be able to synthesize c-di-AMP. Until now, c-di-AMP has only been described as a naturally occurring molecule in the supernatant of *Listeria monocytogenes* cultures and in two very recent studies in the cytoplasm of *B. subtilis* and *Streptococcus pyogenes* [40], [41], [42]. The c-di-AMP in the supernatant of *L. monocytogenes* was shown to be involved in the activation of an IFN-β-mediated host immune response [40]. In the same study, a *L. monocytogenes* protein thought to be essential for bacterial growth and containing a so called DisA_N or DAC domain (Pfam02457) was implicated in the production of c-di-AMP and termed DacA for diadenylate cyclase A [40]. The *S. aureus* membrane protein SAOUHSC_02407 is homologous to this *L. monocytogenes* protein and the only *S. aureus* protein that contains a DisA_N domain. Of note, this gene is also one of the five genes found to contain mutations within two of the SEJ1Δ*ltaS* suppressor strains (Table 1).

**Figure 7:**
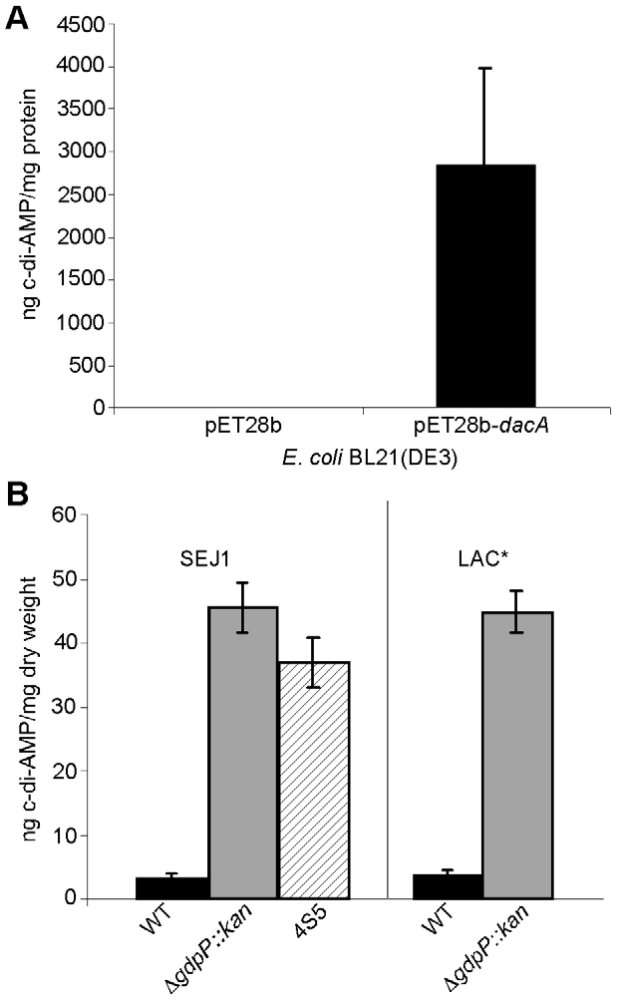
Intracellular c-di-AMP detection. (A) Intracellular c-di-AMP concentration of *E. coli* strains BL21(DE3) harboring the empty vector pET28b or the DacA expression vector pET28b-*dacA*. *E. coli* strains were grown and cell extracts prepared as described in the materials and methods section. c-di-AMP concentrations were determined by HPLC-MS/MS analysis and the average and standard deviation of three values plotted as ng c-di-AMP/mg *E. coli* protein. (B) The concentration of c-di-AMP in the cytoplasm of WT strains SEJ1 and LAC* (black bars), the *gdpP* mutant strains SEJ1Δ*gdpP::kan* and LAC*Δ*gdpP::kan* (grey bars) and the SEJ1-derived suppressor strain 4S5 (striped bar) were quantified by HPLC-MS/MS. Cyclic dinucleotide concentrations are presented as ng c-di-AMP/mg bacterial dry weight and the average and standard deviation of three values are plotted.

To investigate if SAOUHSC_02407 is a c-di-AMP cyclase, this gene was cloned and expressed in *E. coli*, which cannot naturally produce c-di-AMP. *E. coli* extracts were prepared and analyzed by LC-MS/MS as previously described for the detection of c-di-GMP [43]. While no c-di-AMP was detected in *E. coli* extracts isolated from a strain containing the empty plasmid pET28b, extremely high levels of more than 2800 ng c-di-AMP/mg *E. coli* protein were detected in extracts isolated from the strain expressing SAOUHSC_02407 (Figure 7A). This provides experimental evidence that the *S. aureus* protein SAOUHSC_02407, renamed DacA, is a c-di-AMP cyclase and that *S. aureus* should be capable of producing c-di-AMP.

### Inactivation of GdpP results in increased intracellular c-di-AMP levels

To address experimentally if c-di-AMP is produced by *S. aureus* and to investigate the involvement of GdpP in adjusting nucleotide levels, cytoplasmic extracts were prepared from cultures of strains SEJ1 and the *gdpP* mutant SEJ1Δ*gdpP::kan.* C-di-AMP could be readily detected in samples isolated from both strains using an LC-MS/MS method [43]. To accurately quantify c-di-AMP levels, samples were spiked before extraction with a ^13^C^15^N isotope-labeled version of c-di-AMP of known concentration and amounts were quantified based on a c-di-AMP standard curve (Figure S5 in Text S1). A c-di-AMP concentration of 3.33±0.44 ng/mg bacterial dry weight was determined for strain SEJ1 and upon deletion of *gdpP* the c-di-AMP levels increased more than 13-fold to 45.37±3.83 ng/mg bacterial dry weight (Figure 7B). An increase in c-di-AMP concentration of a similar magnitude was also observed when samples were prepared from the LTA-negative suppressor strain 4S5 (Figure 7B). Furthermore, c-di-AMP was also detected in cytoplasmic extracts of the CA-MRSA strain LAC* and again the c-di-AMP levels increased more than 11-fold in the isogenic *gdpP* deletion strain LAC*Δ*gdpP::kan* (Figure 7B). These results establish for the first time the presence of the cyclic dinucleotide c-di-AMP in *S. aureus*, and provide direct evidence that the *S. aureus* GdpP enzyme is a c-di-AMP specific phosphodiesterase *in vivo*. In addition, these results demonstrate that *S. aureus* strains that lack LTA respond by increasing cellular levels of the secondary messenger c-di-AMP. It is also important to note that no quantifiable amounts of c-di-GMP could be detected in *S. aureus* extracts as judged by LC-MS/MS. This is consistent with the findings by Holland *et al*. [44], that GdpS (GGDEF domain protein from *Staphylococcus*), the only staphylococcal protein with a potentially intact GGDEF domain, is unable to synthesize c-di-GMP. Taken together, our findings indicate that c-di-GMP is absent in *S. aureus* but underscores the importance of c-di-AMP as a secondary messenger in *S. aureus*.

### Increased c-di-AMP levels influence cell size and envelope characteristics

To examine whether a deletion of *gdpP,* and the concomitant increase in c-di-AMP, directly affects cell wall properties and could in that way compensate for the lack of LTA, the cell wall characteristics of the mutant strains were examined. Firstly the level of hydrolytic enzymes present on the cell surface of LAC* and the isogenic *gdpP* mutant was analyzed. The *gdpP* mutant strain possessed increased amounts of autolysins as judged by zymographic analysis (Figure S6 in Text S1).

**Figure 8:**
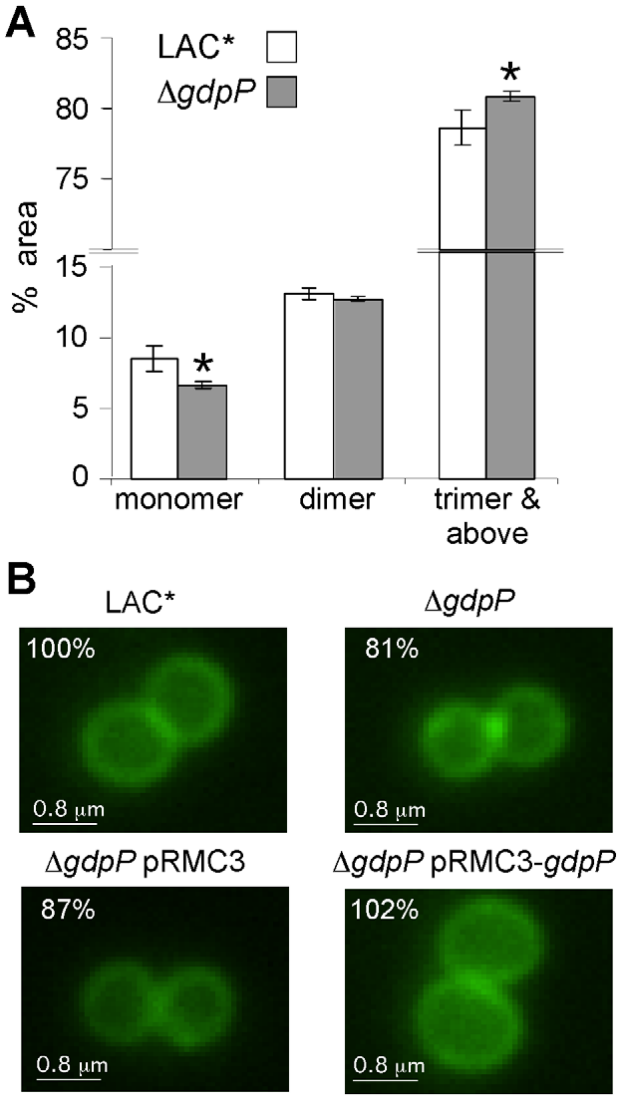
Peptidoglycan and microscopic analysis of LAC* and LAC*Δ*gdpP::kan* strains. (A) Peptidoglycan analysis and quantification of muropeptides. Peptidoglycan was purified from strains LAC* and LAC*Δ*gdpP::kan*, digested with mutanolysin and muropeptides separated by HPLC as described in the materials and methods section. Monomer, dimer and trimer & above peak areas as highlighted in Figures S7A and S7B in Text S1 were integrated and quantified using the Agilent Technology ChemStation software and are shown as percentages of the total peak area (minutes 20 and 145). The average values and standard deviations from three experiments are plotted. For statistical analysis, a two-tailed two sample equal variance Student's *t*-test was performed and statistically significant differences with *p*-values below 0.05 are indicated with an asterisk (*). (B) Microscopic analysis of LAC*, LAC*Δ*gdpP::kan,* LAC*Δ*gdpP::kan* pRMC3 and LAC*Δ*gdpP::kan* pRMC3-*gdpP*. All strains were grown to log-phase in TSB medium containing 100 ng/ml Atet, (plus chloramphenicol for plasmid-containing strains). Bacteria were stained with BODIPY-vancomycin and prepared for microscopy analysis as described in the materials and methods section. The diameters of 100 bacteria per strain were measured in three independent experiments using the Improvision Volocity software. The average diameter for WT LAC* bacteria was set to 100% and the diameters for the other strains are displayed as% of the WT. Actual values of average diameters and standard deviation are listed in Table 3. Larger fields of view are shown in Figure S7C in Text S1.

Next LAC* and the isogenic LAC*Δ*gdpP*::*kan* mutant strain were incubated with increasing concentrations of the cell wall or membrane targeting antimicrobials oxacillin, penicillin G, vancomycin, lysostaphin, daptomycin and nisin. LAC*Δ*gdpP*::*kan* displayed an approximately eight-fold decreased susceptibility to oxacillin and lysostaphin and a more than 32-fold decreased susceptibility to penicillin G (Table 2), indicating that changes in the cell wall/peptidoglycan structure have occurred. To investigate this further, peptidoglycan was purified from the WT and the *gdpP* mutant LAC* strains, digested with mutanolysin and the muropeptides analyzed by HPLC. The overall muropeptide profile of the two strains was very similar (Figure S7A and B in Text S1), however a statistically significant reduction in the amount of monomeric muropeptides and a concurrent increase in higher cross-linked muropeptides (trimers and above) was observed in the *gdpP* mutant strain (Figure 8A). This increase in the amount of cross-linked peptidoglycan could potentially be responsible for the increased resistance to cell wall targeting antimicrobials observed in this strain and could aid with bacterial survival in the absence of LTA by strengthening the cell wall, however this is speculative and remains to be confirmed.

**Table 2:** MIC for different antibiotics in µg/ml for LAC* and LAC*Δ*gdpP*::*kan.*

	LAC*	LAC* Δ*gdpP*	Mode of action
**Lysostaphin**	0.25	2–4	Peptidoglycan – cleaves pentaglycine crossbridges
**Oxacillin**	32–64	256	Peptidoglycan – inhibits PBPs and peptidoglycan crosslinking
**Penicillin G**	0.2	>6.4	Peptidoglycan – inhibits PBPs and peptidoglycan crosslinking
**Vancomycin**	4	4	Peptidoglycan – binds to D-Ala D-Ala and prevents peptidoglycan crosslinking
**Nisin**	6.25–12.5	6.25–12.5	Antimicrobial peptide
**Daptomycin**	4–8	4	Membrane depolarization/disruption

MICs are defined as antibiotic concentration that leads to >75% growth inhibition as compared to growth without antibiotic.

Following this, the WT LAC* strain and the isogenic LAC*Δ*gdpP*::*kan* strain were grown to mid-log phase, stained with BODIPY-vancomycin and observed under the microscope. This analysis revealed a reduction of cell size by more than 13% for the *gdpP* mutant, which could be restored to the WT cell size upon complementation with *gdpP* (Figure 8B and S7C in Text S1). Based on this microscopic analysis, average diameters of 1.188±0.025 µm and 0.967±0.015 µm were determined for the LAC* and LAC*Δ*gdpP::kan* strains, respectively (Table 3). A similar reduction in cell size was observed for the *gdpP* mutant in the RN4220 strain background, where cell diameters of 1.138±0.081 µm for SEJ1 and 0.879±0.051 µm for the SEJ1Δ*gdpP::kan* strain were measured. These results indicate a function for c-di-AMP in controlling the cell size of *S. aureus* with increased cyclic dinucleotide levels leading to a statistically significant decrease in cell size.

Based on the experimentally determined cell diameters and CFU counts for cultures used to prepare the cytoplasmic extracts to detect c-di-AMP (see above Figure 7B) intracellular cyclic dinucleotide concentrations of 2.8±0.6 µM for SEJ1 and 2.1±0.3 µM for the LAC* could be calculated, which increased approximately 15-fold to 42.9±9.0 and 31.5±4.5 µM in the isogenic *gdpP* mutant strains (Table 3).

**Table 3:** Intracellular c-di-AMP concentration in different *S. aureus* strains.

Strain	Sample number	ng c-di-AMP/ml culture	CFU/ml culture	Average cell diameter (µm)	Average intracellular c-di-AMP conc. (µM)
**SEJ1**	**1**	2.524	1.51E+09	1.138±0.081	2.8±0.6
	**2**	2.036	1.34E+09		
	**3**	2.124	2.00E+09		
**SEJ1Δ** ***gdpP::kan***	**1**	29.32	2.36E+09	0.879±0.051	42.9±9.0
	**2**	26.56	2.90E+09		
	**3**	31.44	3.71E+09		
**4S5**	**1**	18.72	1.19E+09	1.141±0.083	29.3±1.4
	**2**	19.44	1.36E+09		
	**3**	18.72	1.26E+09		
LAC*	**1**	2.864	2.11E+09	1.188±0.025	2.1±0.3
	**2**	2.344	1.98E+09		
	**3**	2.376	2.34E+09		
LAC*Δ ***gdpP::kan***	**1**	30.64	3.05E+09	0.967±0.015	31.5±4.5
	**2**	25.12	2.27E+09		
	**3**	27.32	3.28E+09		

The molecular weight of c-di-AMP (free acid) is 658.4 g/l and the volume of a bacterial cell was calculated using 4/3 π r^3^. The diameter of *S. aureus* cells was determined experimentally by microscopic analysis and measuring the diameters of 100 cells each in three independent experiments.

### Like c-di-GMP, increased levels of c-di-AMP affect the ability of *S. aureus* to form a biofilm

In a variety of bacteria an increase in intracellular levels of the related and well-characterized cyclic dinucleotide c-di-GMP, stimulates the biosynthesis of adhesins, promotes biofilm formation and inhibits various forms of motility [45]. No such functions have yet been ascribed to c-di-AMP.

To investigate whether cellular levels of c-di-AMP affect the ability of *S. aureus* to form a biofilm, cultures of WT SEJ1 and both the silent *gdpP* mutant SEJ1Δ*gdpP,* and the marked *gdpP* mutant SEJ1Δ*gdpP::kan,* were grown without shaking in 96-well plates containing BHI 4% NaCl. Staining of adherent bacteria with crystal violet revealed that the *gdpP* deletion strains formed approximately 3-times more biofilm than the WT control strain (Figure S8 in Text S1). However, it should be noted that neither the WT nor the *gdpP* mutant LAC* strains formed robust biofilms under these conditions. Nevertheless, this indicates that increased cellular levels of c-di-AMP not only affect cell properties that allow bacteria to grow in the absence of LTA but, like c-di-GMP, also influence the production of components involved in biofilm formation at least in some *S. aureus* background strains.

## Discussion

**Figure 9:**
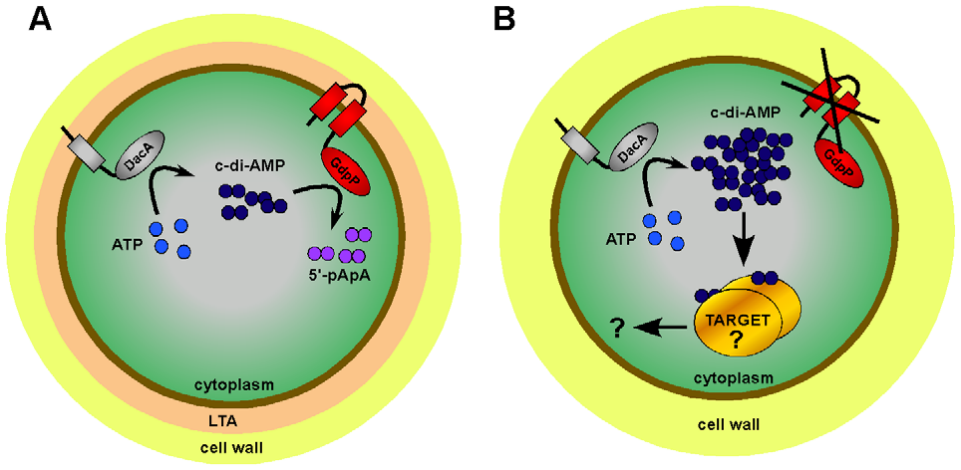
Model of c-di-AMP synthesis and degradation in *S. aureus.* (A) *S. aureus* DacA (grey protein) synthesizes c-di-AMP (dark blue circles) from ATP (light blue circles) and GdpP (red protein) hydrolyses c-di-AMP into 5′-pApA (purple circles). This results in WT LTA-containing (depicted as peach zone) *S. aureus* cells having an intracellular c-di-AMP concentration of approximately 2 to 3 µM. (B) LTA-negative *S. aureus* suppressor strains survive by inactivating the phosphodiesterase activity of GdpP resulting in an increase in intracellular c-di-AMP levels. Next, it is assumed that at this increased concentration c-di-AMP binds to a specific set of target proteins and either directly affects their activity or indirectly affects the expression of other proteins, altogether compensating for the cell wall defect caused by the absence of LTA.

Clear functions for the secondary messenger molecule c-di-GMP, in controlling gene expression and the switch from planktonic to sedentary lifestyles, have been established in a diverse range of bacterial species [45]. It is now well documented that this cyclic dinucleotide plays an important role in controlling biofilm formation and virulence gene expression in a range of bacteria, including important human pathogens such as *Pseudomonas aeruginosa* [46]. Recently, it has also been suggested that this signaling molecule, which is widespread in bacterial species but apparently not found in higher eukaryotes, can act as a danger signal in eukaryotic cells prompting studies on the immunomodulatory and immunostimulatory properties of c-di-GMP [47], [48]. On the other hand, until very recently c-di-AMP had not been recognized as a naturally occurring molecule in any living organism. c-di-AMP was noticed for the first time in 2008 during crystallization studies of the *B. subtilis* DNA binding protein DisA [49], [50]. Additional work confirmed that the N-terminal part of DisA (DisA_N domain) is capable of synthesizing c-di-AMP *in vitro* from two molecules of ATP [49]. Shortly afterwards, c-di-AMP phosphodiesterase activity was ascribed to the *B. subtilis* protein YybT using an *in vitro* assay system [39]. The first evidence for the production of c-di-AMP by living cells came from a study on *L. monocytogenes*, where this cyclic dinucleotide was detected in the culture supernatant and identified as the molecule that stimulates an IFN-β-mediated host immune response [40]. Very recently c-di-AMP has also been detected in cytoplasmic extracts from *S. pyogenes* and from *B. subtilis* [41], [42]. In this study, we have identified c-di-AMP within the cytoplasm of the Gram-positive pathogen *S. aureus* and also quantified the amounts produced *in vivo* in both laboratory and clinically relevant strains (Figure 7 and Table 3). Intracellular c-di-AMP concentrations of 2 to 3 µM were detected in the *S. aureus* cells, which are very similar to the concentration of 1.7 µM reported for *B. subtilis* during vegetative growth [41]. We also show in this study that GdpP functions *in vivo* as a c-di-AMP-specific phosphodiesterase and provide experimental evidence that the *S. aureus* protein SAOUHSC_02407, which was renamed DacA, is capable of producing c-di-AMP (Figure 7 and 9). Similar to what was observed with the c-di-GMP signaling molecule, this study provides a link between this novel cyclic nucleotide and cell wall properties in Gram-positive bacteria, as an increase in c-di-AMP levels allows *S. aureus* to grow in the absence of LTA and the CA-MRSA *gdpP* mutant strain shows an increased resistance to the cell wall active antimicrobials oxacillin, penicillin G and lysostaphin (Table 2) and an increase in the amount of cross-linked peptidoglycan (Figure 8A). Our study also revealed that c-di-AMP plays a role in controlling the cell size of *S. aureus* (Figure 8B). Recently, Oppenheimer-Shaanan *et. al* have shown that c-di-AMP levels increase 3-fold in *B. subtilis* at the onset of sporulation and the authors suggested that this increase serves as a positive signal for sporulation to proceed [41]. Since *S. aureus* does not form spores, this particular function attributed to c-di-AMP cannot apply to *S. aureus*. However, our observation that an increase in c-di-AMP levels results in a decrease in cell size suggests perhaps a more general function for c-di-AMP in progressing the cell cycle and not just the sporulation process in Gram-positive bacteria. In this respect, it is interesting to note that, in contrast to c-di-GMP, c-di-AMP might be an essential constituent of the cell. Attempts to disrupt the c-di-AMP cyclase DacA in *L. monocytogenes* were unsuccessful [40]. Furthermore, screens for essential genes in *Mycoplasma pulmonis*, *Mycoplasma genitalium*, *Streptococcus pneumoniae* as well as *S. aureus* indicated that *dacA* is essential for cell viability [51], [52], [53], [54].

The c-di-AMP cyclase DacA and the c-di-AMP phosphodiesterase GdpP are both predicted to be anchored to the bacterial membrane and it seems likely that changes in the environment, cell wall structures or in the membrane itself serve as cues to adjust the intracellular cyclic dinucleotide levels. Bioinformatic analysis of GdpP revealed that this protein contains a highly degenerated PAS domain. PAS domains usually function as sensory domains for detecting light, redox potential, oxygen, small ligands, and the overall energy level of a cell, usually by way of an associated cofactor [55]. A recent study has shown that the PAS domain of the *B. subtilis* YybT protein is capable of binding the cofactor heme and that this binding suppresses the phosphodiesterase activity *in vitro* [56]. This indicates that the PAS domain of YybT is indeed capable of sensing environmental changes and the most likely output will be a change in c-di-AMP levels. In this regard it is interesting to note that we observed a light brown discoloration of the Ni-NTA columns during the purification process of the GdpP protein, suggesting that the *S. aureus* protein is also capable of binding heme.

GGDEF domains are typically associated with c-di-GMP cyclases or phosphodiesterases and function to synthesize c-di-GMP. Degenerated domains can also regulate the activity of associated c-di-GMP phosphodiesterase domains [57]. The GGDEF domain of GdpP has the highly divergent amino acid sequence SSDQF, with substitutions of three of the five highly conserved active site residues that are essential for GTP catalysis [58]. The GGDEF domain of *B. subtilis* YybT can bind ATP and slowly convert it to ADP [39]. However, this domain cannot synthesize c-di-AMP and the ATP binding had no effect on the *in vitro* phosphodiesterase activity contained within the DHH/DHHA1 domains [39]. The biological relevance of the ATP binding and hydrolysis activity of the degenerated GGDEF domain remains to be determined. Indeed, we provide experimental evidence in this study that argues against a role of any potential ATPase activity for the function of the *S. aureus* GdpP protein (Figure 6). At the same time our data suggest that this domain can influence, independently of any ATPase activity, the phosphodiesterase activity of the associated DHH/DHHA1 domains, as indicated by a decrease in *in vitro* phosphodiesterase activity of GdpP variants containing single point mutations in the GGDEF domain (Figure 6). Additional experiments are needed to determine the mechanism by which the GGDEF domain influences the activity of the downstream phosphodiesterase.

As mentioned above, c-di-GMP is typically synthesized by GGDEF domain containing proteins and a search for this domain highlighted two *S. aureus* proteins that could potentially function as c-di-AMP cyclases. GdpP itself, which contains amino acid alternations in crucial resides in this domain and extrapolating from the rigorous *in vitro* analysis on the *B. subtilis* homologue YybT, does not have cyclase activity. The second protein is GdpS [44]. O'Gara and coworkers investigated the possibility that the *Staphylococcus epidermidis* GdpS protein is a c-di-GMP cyclase but with no success and consistent with this observation we show in this study that c-di-GMP does not appear to be present in *S. aureus* [44]. It is possible however, that GdpS may be involved in c-di-AMP synthesis instead, a theory that is currently under investigation. In this regard it is interesting to note that, in contrast to the *gdpP* deletion strain analyzed in this study that causes an increase in biofilm formation (Figure S8 in Text S1), an *S. epidermidis gdpS* mutant strain has a defect in biofilm formation caused by a decrease in transcription of the polysaccharide-producing *ica* locus [44].

While the exact number of *S. aureus* proteins involved in c-di-AMP production and hydrolysis remains to be determined, it is certain that GdpP and DacA are involved in this process (Figure 7). Having shown that a deletion of *gdpP* results in increased intracellular levels of c-di-AMP the question remains as to how this suppresses the bacterial need for LTA. Rao *et al.*, have demonstrated that the alarmone ppGpp, which is produced during the stringent response to cope with stress, inhibits the phosphodiesterase activity of YybT *in vitro* [39]. Furthermore, disruption of the GdpP homologues in *Lactococcus lactis* and *B. subtilis* renders these bacteria more resistant to acid stress [39], [59]. The deletion in *B. subtilis* also increases the sporulation efficiency of cells that had been exposed to a DNA damaging agent [39], [41]. All these observations indicate that an increase in intracellular c-di-AMP levels allows bacteria to cope better under stress conditions. Undoubtedly depleting LTA from the cell wall will place bacteria under stress. While bacteria may not naturally be able to respond to such a stress, one mechanism that allows bacteria to survive is by irreversibly disrupting the function of GdpP and in this manner increasing intracellular c-di-AMP levels (Figure 7B). By analogy with c-di-GMP, we assume that c-di-AMP then acts as a secondary messenger to up- or down regulate the activity or expression of a certain set of target proteins (Figure 9) [45]. Alternatively, c-di-AMP might also bind to RNA molecules to affect protein expression, as in the case of c-di-GMP-dependent riboswitches [60]. As an increase in c-di-AMP concentration in LTA depleted cells has served to improve cell growth and division (Figure 3) it is tempting to speculate that c-di-AMP is involved in regulating some components of the cell division machinery. A function for c-di-AMP in controlling cell division in *S. aureus* is also consistent with our observations that *gdpP* mutant strains are smaller in size (Figure 8B and Table 3). Therefore, an increase in intracellular c-di-AMP appears to allow *S. aureus* cells to initiate cell division before they have reached their normal size. Furthermore, the increased resistance of a *gdpP* mutant LAC* strain to lysostaphin, oxacillin and penicillin G and the decrease in monomeric peptidoglycan subunits provides experimental evidence that proteins involved in peptidoglycan synthesis are regulated by c-di-AMP. It was also noted that three LAC* suppressor strains contained WT *gdpP* sequences (Table S2 in Text S1). It is possible that in these strains the molecular targets of c-di-AMP are mutated instead of altering the concentration of the second messenger molecule. While beyond the scope of this study, it will be interesting to identify c-di-AMP target proteins in future studies, in particular those involved in helping the bacteria cope with stress. One could suppose that by interfering with c-di-AMP synthesis cells may be less able to respond and cope with the stresses encountered during infection. By identifying a way to combine this inhibition with currently available drug treatments, it could be possible to control even the most multi-drug resistant *S. aureus* infections.

Inactivation of GdpP was the first step that allowed all five sequenced suppressor strains to grow in the absence of LTA, however several of the mutations acquired in subsequent steps are interesting to note. Two suppressor strains acquired mutations that lead to amino acid substitutions in the c-di-AMP cyclase DacA (Table 1). These substitutions might function to improve cyclase activity and, analogous to a *gdpP* deletion, this may result in an overall increase in c-di-AMP levels. It is also plausible that these mutations function to reduce the drastically increased levels of c-di-AMP observed in suppressor strains, levels which could prove toxic if not regulated properly. A third suppressor mutation resulted in an amino acid substitution in the succinate dehydrogenase SdhA (SAOUHSC_01104). SdhA is a TCA cycle enzyme and it is hard to predict how alterations in a central metabolism enzyme could help LTA-deficient bacteria to survive. The remaining two mutated genes, encode for a putative permease (SAOUHSC_01358) and a conserved hypothetical protein with sequence homology to a fusaric acid transporter (SAOUHSC_02001) (Table 1). Premature stop codons have arisen within these two proteins presumably inactivating their function. Woodward *et al* have shown that over-expression of multidrug resistance transporters results in increased secretion of c-di-AMP into the supernatant of *L. monocytogenes* cultures [40]. It is therefore plausible that disrupting the functions of these two putative *S. aureus* transporters may again serve to increase intracellular c-di-AMP levels.

Eukaryotic host cells often recognize essential bacterial cell components as a means of detecting an infection. One well-studied example of this is the recognition of peptidoglycan by the intracellular host proteins Nod1 and Nod2, which upon detection results in the activation of the NF-κΒ pathway and an immune response [61]. Recently, Woodward and coworkers discovered that c-di-AMP, which is released by *L. monocytogenes* inside host cells, is also detected in the cytosol where it triggers a host immune response [40]. *S. aureus* is another pathogen that is capable of invading epithelial and endothelial cells through fibronectin binding protein-mediated adherence to the host cell integrin α5β1 and subsequent endocytosis [62]. Once inside the cell it is equally likely that *S. aureus* secretes this potentially essential nucleotide into the cytosol, a possibility that may then be exploited by the host immune system. The eukaryotic proteins involved in the detection of c-di-AMP and the mechanism leading to immune activation remains to be determined as our current understanding is only rudimentary [63].

Taken together we have shown that it is possible to create viable LTA-negative strains of *S. aureus* that compensate for the loss of this important polymer by increasing intracellular levels of the secondary messenger c-di-AMP. The unambivalent identification of c-di-AMP in the cytoplasm of *S. aureus* and the ability to regulate its level opens up the exciting possibility of identifying target proteins or other compounds through which this cyclic dinucleotide exerts its function and regulates cellular processes. This is especially intriguing for Gram-positive pathogens such as *S. aureus, S. pneumonia, S. pyogenes* and *L. monocytogenes* considering that c-di-AMP may be essential for cell viability. Further studies are required to fully elucidate the role of this messenger in the cell and to ultimately discover what targets are altered in response to a deletion of LTA. This will hopefully help us to more fully understand the biological significance of this polymer and to identify novel essential cellular survival mechanisms that could be exploited as therapeutic drug targets.

## Materials and Methods

### Bacterial strains and culture conditions

Strains used in this study are listed in Table S3 in Text S1 and primers used for cloning in Table S4 in Text S1. *E. coli* and *B. subtilis* strains were grown in LB and *S. aureus* strains were grown in TSB medium at 37°C with aeration, if not otherwise stated. When required, media were supplemented with antibiotics and inducers as indicated in Table S3 in Text S1. Details on plasmid and strain constructions are provided in the supplementary Materials and Methods section in Text S1.

### Growth curves and determination of CFUs

WT and LTA suppressor strains were grown overnight in TSB medium. Unsuppressed Δ*ltaS* strains were grown in TSB containing either 7.5% NaCl or 40% sucrose. Overnight cultures were washed three times in the appropriate medium and diluted to a starting OD_600_ of 0.05. Cultures were incubated at 37°C with aeration and OD_600_ values determined at 2 h intervals. Cultures containing *ltaS* under IPTG inducible control were grown overnight in the presence of IPTG and the appropriate antibiotics. Bacteria were washed three times in TSB and diluted to an OD_600_ of 0.05 in 5 ml TSB with or without 1 mM IPTG and 100 ng/ml Atet as appropriate and OD_600_ values determined. Where stated, the cultures were diluted after 4 h 1∶100 into fresh medium with the appropriate antibiotics and inducers to maintain cultures in the exponential growth phase and growth continued for a further 6 h. The 4 h time point is then represented as T = 0. Growth curves were performed in triplicate and representative graphs are shown. CFUs per ml culture were determined by emulsifying a colony in 1 ml TSB, normalizing the OD_600_ to 0.05, performing serial dilutions and plating 100 µl on the appropriate plates. Plates were then incubated at 30°C or 37°C as indicated and colonies were enumerated after overnight growth. Counts were performed twice with representative figures stated in the text.

### Whole genome sequencing

For whole genome sequence determination, chromosomal DNA was isolated from the *S. aureus* reference strain SEJ1 and the 5 suppressor strains 4S4, 4S5, 4N1, 4N2 and 5S4. Sequences were determined using a SOLiD 3 System (Applied Biosciences) and DNA fragment libraries. Fifty bp fragment libraries were generated by mechanical shearing, treated and coupled to beads using emulsion PCR and deposited on glass slides following protocols supplied by Applied Biosciences. The generated reads resulted in genomic coverage of between 140x and 213x and have been submitted to the ENA Sequence Read Archive (SRA) under accession ERP000528 (http://www.ebi.ac.uk/ena/data/view/ERP000528). Reads from SEJ1 were aligned to the known genome sequence of *S. aureus* strain NCTC8325 with the BioScope mapping pipeline (Applied Biosystems) using an anchor length of 25 bp allowing 2 mismatches. Next the reads from the suppressor strains were individually aligned to NCTC8325 using the same alignment procedure. Using these analysis tools, large DNA deletions in place of the prophage sequences and the *spa* gene as well as 84 point mutations with high confidence scores were detected in all strains (SEJ1 and the 5 suppressor strains) when compared to the NCTC8325 genome sequence. Of these 84 mutations, 76 were identical to those listed in the recently published paper on the RN4220 genome sequence [34]. Of the 8 that differed, 7 were located in a highly repetitive genome region and are likely to represent misalignments of reads. Of the extra 45 snps identified by Nair *et. al.,* [34], a number are caused by insertion or deletion events, mutations we could not detect because of limitations of the analysis software and due to the use of a fragment library rather than using mate-pairs. Ten additional mutations, identified using the BioScope diBayes pipeline (Applied Biosystems) and ‘high’ call stringency setting, were present in the suppressors strains but absent in strain SEJ1 and these mutations are described in detail in the results section. Suppressor strains specific differences were verified by re-sequencing the genes in question at the MRC Clinical Science Centre Sequencing Facility at Imperial College London.

### Western blot, autolysis assays, zymogram assays, biofilm assays, minimum inhibitory concentrations, phase contrast and fluorescence microscopy

These assays were performed using standard procedures and details can be found in the supplementary Materials and Methods section in Text S1.

### Protein purification

His-tagged *S. aureus* GdpP protein variants and the *B. subtilis* DisA protein were purified from 1 to 2 liter induced *E. coli* BL21(DE3) cultures containing the respective pET28b expression vectors (Table S3 in Text S1). Protein induction and purification was performed as described in Rao *et al.* with minor modifications and details can be found in Text S1 [39].

### *In vitro* c-di-AMP phosphodiesterase activity assay

The enzymatic activities of the different purified GdpP protein variants was determined by incubating 20 µM c-di-AMP (BioLog) with 1 µM purified protein in buffer containing 50 mM Tris pH 8.5, 20 mM KCl and 0.1 mM MnCl_2_. Enzyme reactions were stopped at the indicated time by the addition of an equal volume of 0.1 M EDTA pH 8 and incubation for 3 to 5 min at 95°C. Enzymatic activity was determined by separating 15 µl of the reaction mixtures by HPLC (Agilent LC1200) using a Luna 150×2, 3 µm particle size RP C-18 column and a 0.1 M triethylamine acetic acid pH 6.1 (Buffer A) and 80% acetonitrile containing 20% buffer A (Buffer B) solvent system. The column temperature was set to 35°C and the flow rate to 0.25 ml/min and a constant buffer B concentration of 5% was used for the runs. Nucleotides were detected at A_254_ and authentic c-di-AMP and 5′-pApA (BioLog) were used as standards to determine nucleotide specific retention times.% c-di-AMP hydrolysis was calculated based on integrated nucleotide peak areas. Four independent experiments were performed (using proteins from two separate purifications) and the average and standard deviation of all four values is plotted.

### Preparation of bacterial extracts for c-di-AMP measurements

Overnight cultures of *S. aureus* cells were diluted to a starting OD_600_ of 0.05 and grown for 4 h at 37°C with aeration. Cultures were adjusted to an approximate OD_600_ of 2, CFU counts determined by plating appropriate dilutions on TSA plates and bacteria from a 10 ml culture aliquot were also collected by centrifugation, washed and freeze dried to determine the dry weight, for normalization purposes. A 5 ml aliquot from the same culture was removed and bacteria collected by centrifugation at 9,000×*g* for 5 min. The pellet was suspended in 1 ml ice-cold extraction buffer (acetonitrile/MeOH/H_2_O - 40∶40∶20; LC-MS gradient grade, VWR) containing 0.58 µM internal ^13^C^15^N isotope labeled c-di-AMP standard. Samples were snap frozen with liquid N_2_ for 15 sec before being heated to 95°C for 10 min. Samples were mixed with 0.5 ml of 0.1 mm glass beads and lysed in a Fast-Prep machine 2 x for 45 sec at setting 6 (FP120, MP Biomedicals, LLC). Glass beads were separated by centrifugation at 17,000×*g* for 5 min at 4°C. The supernatant was removed and stored at 4°C and the remaining glass beads/cell debris mixture was washed with 1 ml extraction buffer without internal standard, incubated on ice for 15 min and again lysed. Samples were once again spun and the supernatant combined with the previous one. Glass beads were washed with 1 ml extraction buffer, incubated on ice for 15 min and centrifuged. All supernatants were combined and samples dried at 40°C under a stream of N_2_ and stored at −80°C. *E. coli* overnight cultures were diluted 1∶100 into fresh LB medium and grown at 37°C to an OD_600_ of 0.5 at which point 1 mM IPTG was added and cultures were grown for a further 3 h. For normalization purposes, culture aliquots corresponding to an OD_600_ of 2 were withdrawn, washed once in PBS pH 7.4, suspended in 800 µl 0.1 M NaOH and heated to 95°C for 15 min. The samples were centrifuged for 5 min at 17,000×*g* and the protein content of the supernatant was determined using a BCA assay kit (Pierce). Aliquots corresponding to an OD_600_ of 20 were withdrawn from the same cultures and centrifuged for 5 min at 9,000×*g*. The pellet was suspended in 300 µl ice-cold extraction buffer (acetonitrile/MeOH/H_2_O; 40∶40∶20) containing cXMP (BioLog) as an internal standard at a final concentration of 200 ng/ml. Samples were frozen with liquid nitrogen for 15 sec and afterwards heated to 95°C for 10 min. Samples were centrifuged at 17,000×*g* for 5 min at 4°C and the supernatant removed. The pellet was suspended in 200 µl extraction buffer without cXMP, incubated on ice for 15 min and centrifuged. The supernatant was combined with the previous one and the pellet was once again suspended in 200 µl extraction buffer followed by a 15 min incubation on ice and a centrifugation step. All supernatants were combined and dried at 40°C under a flow of N_2_. The c-di-AMP concentration was determined based on a c-di-AMP standard curve and values are presented as ng c-di-AMP/mg *E. coli* protein.

### Preparation of isotope labeled c-di-AMP

For the synthesis of ^13^C^15^N isotope labeled c-di-AMP, 1 µM rDisA was incubated with 500 µM adenosine-^13^C^15^N-5′-triphosphate in 10 mM Tris-HCl, pH 7.5, 10 mM MgCl_2_ and 0.1% BSA for 18 h at 30°C with gentle mixing (300 rpm). Based on an identical experiment using unlabeled ATP as the substrate, it was deduced that substrate turnover is complete under these conditions. The reaction was stopped by heating samples to 95°C for 15 min and the suspension was clarified by centrifugation (20,800×*g*; 10 min; 4°C). The concentration of ^13^C^15^N-c-di-AMP in the supernatant was determined by measuring the absorption at 259 nm (ε_259 nm_ = 30,000 M^−1^cm^−1^). Further purification steps were not necessary for the use of ^13^C^15^N-c-di-AMP as an internal standard and were therefore omitted.

### Quantification of c-di-AMP by LC-MS/MS

c-di-AMP was detected and quantified by LC-MS/MS using a similar protocol as published previously for the detection of c-di-GMP [43]. Details are given in the supplementary Materials and Methods section in Text S1.

### Muropeptide analysis by HPLC

For muropeptide analysis, 1 liter TSB medium was inoculated with overnight cultures of strains LAC* (AH1263) or LAC*Δ*gdpP::kan* (ANG1961) to an OD_600_ of 0.06. The cultures were grown at 37°C to mid-log phase (OD_600_ of approximately 1.5), cooled on ice and bacteria subsequently collected by centrifugation. Peptidoglycan was purified using a well-established method, which we have used previously [64], [65]. Next, 5 mg purified peptidoglycan was digested in a final volume of 1.24 ml in 12.5 mM phosphate buffer pH 5.9 with 50 µg mutanolysin from *Streptomyces globisporus* (Sigma) for 20 h at 37°C. The suspension was subsequently boiled for 5 min and the insoluble material removed by centrifugation for 5 min at 17,000×*g* and the soluble fraction stored at 4 or −20°C. Immediately before HPLC analysis, a portion of the soluble muropeptide fraction was mixed with an equal volume of 0.5 M Na borate buffer pH 9 and reduced for 30 min at RT by the addition of sodium tetraborohydride. Subsequently the pH was adjusted to 2–3 with 20% phosphoric acid and 100 µl was analyzed by HPLC using a 3 µm-particle-size 120A pore size octyldecyl silane Hypersil 250×4.6 mm C18 column equipped with a 10×4 mm guard column made of the same material (Thermos Electron Corporation). The column temperature was set to 52°C and a sodium phosphate/methanol buffer system and gradient conditions were used as previously described with the exception that the sodium azide was omitted from buffer A [64], [66]. HPLC traces were recorded at 205 nm and the muropeptide profile from three independently grown cultures was determined and a representative graph is shown. For quantification purposes, the area of muropeptide peaks was integrated and quantified using the Agilent Technology ChemStation software and shown as percentage of the total (min 20 and 145) peak area and the average values and standard deviations of the three experiments is given. The two-tailed two sample equal variance Student's *t*-test was used to determine statistically significant differences between muropeptide peak areas and statistically significant differences with *p*-values below 0.05 are indicated with an asterisk (*).

### Gene accession numbers (Swissprot identities)

*S. aureus* NCTC8325: *ltaS* - SAOUHSC_00728; *gdpP* - SAOUHSC_00015; *dacA* - SAOUHSC_02407; *gdpS* - SAOUHSC_00760; gene containing suppressor mutation 2 - SAOUHSC_01104; mutation 3 - SAOUHSC_01358; mutation 4 - SAOUHSC_02001; *atl* – SAOUHSC_00994; *B. subtilis* str.168: *yybT* - BSU40510; *disA* - BSU00880; *L. monocytogenes* str. EGD-e: *dacA –* lmo2120*; Streptococcus pyogenes* SF370: *dacA* - Spy1036

## Supporting Information

Text S1 **Supplementary information**. This file contains a supplementary Materials and Methods section, supplementary Figures S1 to S8 and supplementary Tables S1 to S4.
(DOC)
