# Supplementary material for: c-di-AMP Is a New Second Messenger in Staphylococcus aureus with a Role in Controlling Cell Size and Envelope Stress
Source: PLoS Pathog. 2011 Sep 1;7(9):e1002217. doi: 10.1371/journal.ppat.1002217 (PMC3164647; doi:10.1371/journal.ppat.1002217)
Supplement: Text S1 — Supplementary information. This file contains a supplementary Materials and Methods section, supplementary Figures S1 to S8 and supplementary Tables S1 to S4. (DOC) [file ppat.1002217.s001.doc]

**Text S1**

**Supplementary Materials and Methods**

**Plasmid and strain construction** Strains used in this study are listed in Table S3 and primers used in this study are listed in Table S4. Plasmid pCN34-*ltaS* was created by amplifying the *ltaS* gene and promoter region from pCL55-*ltaS* using primers ANG86/ANG87. The resulting PCR product was digested with BamHI and SalI and cloned into pCN34 that had been digested with the same enzymes. The anhydrotetracycline (Atet) inducible *S. aureus* expression vector pCN34iTET was constructed by amplifying the *xyl/tetO* promoter and *tetR* repressor gene from plasmid pRMC2 using primers ANG908/ANG948. The PCR product was digested with NarI and XmaI and ligated with pCN34 that had been cut with the same enzymes. Plasmids pCN34iTET-*gdpP*, pCN4iTET-*gdpP*_4S4_, pCN34iTET-*gdpP*_4S5_ and pCN34iTET-*gdpP*_4N2_ were constructed by amplifying the *gdpP* gene from genomic DNA isolated from strains SEJ1, 4S4, 4S5 and 4N2, respectively, with primers ANG921/ANG922. The PCR products were digested with KpnI and EcoRI and ligated with pCN34iTET that had been cut with the same enzymes. The plasmids pCN34iTET-*gdpP*_D223A_, pCN34iTET-*gdpP*_R289A_, pCN34iTET-*gdpP*_D418A_ and pCN34iTET-*gdpP*_D497A_ were created by QuikChange site-directed mutagenesis using primer pairs ANG1156/ANG1157, ANG1158/ANG1159, ANG1160/ANG1161 and ANG1162/ANG1163, respectively and using pCN34iTET-*gdpP* as the template in PCR reactions. Plasmids pET28b-*gdpP*_84-655_, for expression of GdpP_84-655_ containing the PAS, GGDEF and DHH/DHHA1 domains, and pET28b-*gdpP*_84-301_, for expression of GdpP_84-301_ containing the PAS and GGDEF domains, were constructed by amplifying the corresponding *gdpP* fragments from SEJ1 genomic DNA using primer pairs ANG1132/ANG922 and ANG1132/ANG1134, respectively. The resulting PCR products were digested with NheI and EcoRI and cloned into pET28b that has been digested with the same enzymes. Plasmids pET28b-*gdpP*_D223A_, pET28b-*gdpP*_R289A_, pET28b-*gdpP*_D418A_, pET28b-*gdpP*_D497A_, pET28b-*gdpP*_4S4_ and pET28b-*gdpP*_4S5_ were created by amplifying the respective *gdpP* alleles from plasmids pCN34iTET-*gdpP*_D223A_, pCN34iTET-*gdpP*_R289A_, pCN34iTET-*gdpP*_D418A_, pCN34iTET-*gdpP*_D497A_, pCN34iTET-*gdpP*_4S4_ and pCN34iTET-*gdpP*_4S5_ with primers ANG1132/ANG922, cutting the PCR products with NheI and EcoRI and cloning them into digested pET28b. Plasmid pET28b-*dacA* was created by amplifying the *dacA* gene from SEJ1 chromosomal DNA using primers ANG1209/ANG1211. The resulting PCR product was digested with NcoI and EcoRI and cloned into pET28b that has been digested with the same enzymes. pET28b-*disA* was created by amplifying the *disA* gene from *B. subtilis* 168 chromosomal DNA using primers ANG1205/ANG1206 and inserting the NdeI and EcoRI cut PCR product into NdeI and EcoRI digested pET28b. Plasmid pCN38iTET was constructed by isolating the chloramphenicol (Cam) cassette from pCN38 following digestion with AvrII and SacII and cloning it into pCN34iTET that has been cut with the same enzymes. This resulted in the replacement of the Kan cassette in pCN34iTET with a Cam marker. To prevent readthrough and ensure plasmid stability two transcription terminators (TT) were cloned into pCN38iTET, one downstream of the multiple cloning site and the other after the *tetR* repressor gene. To this end a TT was amplified from plasmid pCN49 using primers ANG1219/ANG1220, digested with XmaI and SphI and cloned into pCN38iTET that had been cut with the same enzymes. This created plasmid pCN38iTET-TT with a TT after the multiple cloning site. A second TT was amplified, once again from pCN49, this time with the primers ANG1221/ANG1222. This PCR product was digested with NarI and ligated with pCN38iTET-TT that has also been digested with NarI, resulting in the creation of the *S. aureus/E. coli* shuttle vector pRMC3. pRMC3-*gdpP* was constructed by digesting the *gdpP* gene from pCN34iTET-*gdpP* with KpnI and EcoRI and cloning it into the plasmid pRMC3 that has been cut with the same enzymes. All plasmids were initially transformed into *E. coli* strain XL1-Blue and sequences of all inserts were verified by fluorescence automated sequencing at the MRC Clinical Science Centre Sequencing Facility at Imperial College London. For protein expression and purification, all pET28b derived plasmids were transformed into *E. coli* strain BL21(DE3).

For deletion of the *ltaS* gene the method of Oku *et al*., 2009 was used with some modifications . One kb fragments up- and downstream of *ltaS* were amplified from SEJ1 genomic DNA using primers pairs ANG241/ANG671 and ANG669/ANG572, which incorporate 5' and 3' AttB sites, respectively. Purified PCR products were digested with KpnI, ligated and recombined with pKOR1. The resulting plasmid pKOR1-Δ*ltaS* was recovered in *E. coli* strain DH5α and subsequently electroporated into SEJ1 and stably maintained at 30°C in the presence of 10 μg/ml Cam. Shifting the temperature to 43°C resulted in a single cross over event and insertion of the plasmid into the chromosome. Upon confirmation of chromosomal insertion by PCR, the covering plasmid pCN34-*ltaS* was introduced into the strain by electroporation after growth at 37°C in the presence of 5 μg/ml Cam. Growth of the strain at 30°C in the absence of Cam, while selecting for the covering plasmid with 90 μg/ml kanamycin, resulted in excision of pKOR1 and deletion of the chromosomal copy of the *ltaS* gene. Introduction of and selection for pCN38, a plasmid with the same replication of origin as pCN34, resulted in the loss of pCN34-*ltaS* when plated on TSA 7.5% NaCl or 40% sucrose 10 μg/ml Cam plates. The *ltaS* deletion in strains SEJ1Δ*ltaS*_N_ pCN38 (ANG1480; isolated on 7.5% NaCl) and SEJ1Δ*ltaS*_S_ pCN38 (ANG1481; isolated on 40% sucrose) was confirmed by PCR using primer pair ANG247/ANG248. All experiments were performed without selection for pCN38 (no chloramphenicol was added to the medium) and hence strains are referred to as SEJ1Δ*ltaS*_N_ and SEJ1Δ*ltaS*_S_.

For the creation of a marked *ltaS* deletion, the same 1 kb up- and downstream fragments were amplified with primer pairs ANG241/ANG852 and ANG849/ANG572. An ErmAM cassette was amplified from plasmid pMUTIN-HA using primer pair ANG850/ANG851. Purified PCR products were then fused by SOE (Splice Overlap Extension) PCR using primers ANG241/ANG572. The resulting PCR product was again recombined with pKOR1 and transformed into DH5α. pKOR1-Δ*ltaS*::*erm* was electroporated into SEJ1 and deletion of the chromosomal copy of *ltaS* was achieved in the presence of pCN34-*ltaS* as described above. Due to the presence of the Erm marker it was then possible to phage transduce the *ltaS*::*erm* region into the Erm sensitive CA-MRSA strain LAC* pCN34-*ltaS* using Φ11. The incompatible plasmid pCN38 was introduced into strain LAC*Δ*ltaS*::*erm* pCN34-*ltaS* and the complete *ltaS* deletion strains, LAC*Δ*ltaS*_N_::*erm* pCN38 and LAC*Δ*ltaS*_S_::*erm* pCN38, were created as above. All experiments were performed without selection for pCN38 and hence strains are referred to as LAC*Δ*ltaS*_N_::*erm* and LAC*Δ*ltaS*_S_::*erm*.

For the deletion of the *gdpP* gene, 1 kb fragments up- and downstream of *gdpP* were amplified from SEJ1 genomic DNA using primer pairs ANG1054/ANG993 and ANG992/ANG1067, which incorporate 5' KpnI and 3' BamHI sites, respectively. Purified PCR products were then fused by SOE PCR using primers ANG1054/ANG1067, digested with KpnI and BamHI and cloned into the allelic exchange vector pTS1 yielding plasmid pTS1-Δ*gdpP*. This plasmid was then electroporated into SEJ1 and stably maintained at 30°C in the presence of 10 μg/ml Cam. Shifting the temperature to 43°C resulted in insertion of the plasmid into the chromosome. The covering plasmid pCN34iTET-*gdpP* was introduced into the strain, as it was at this point unknown if this gene was essential for growth or not. Downshift of the temperature to 30°C in the absence of Cam but in the presence of Kan90 and Atet200 to select for and induce expression of GdpP from the plasmid, resulted in excision of the pTS1 plasmid and created an inframe deletion of the chromosomal copy of the *gdpP* gene. Introduction and selection for pCN38 resulted in the ready loss of pCN34iTET-*gdpP* on TSA plates and production of the *gdpP* deletion strain SEJ1Δ*gdpP* pCN38. When experiments were performed without selection for plasmid pCN38 this strain is referred to as SEJ1Δ*gdpP*.

For the creation of a marked *gdpP* deletion, 1 kb up- and downstream fragments were amplified with primer pairs ANG990/ANG1166 and ANG1169/ANG991. A kanamycin cassette was amplified from plasmid pCN34 using primer pair ANG1167/ANG1168. Purified PCR products were then fused by SOE PCR using primers ANG946/ANG947, which also added AttB sites for recombination with pKOR1. The resulting PCR product was recombined with pKOR1 and transformed into DH5α. pKOR1-Δ*gdpP*::*kan* was then electroporated into SEJ1 and allelic exchange performed as previously described resulting in strain SEJ1Δ*gdpP*::*kan* (ANG1958). Replacement of the gene was confirmed using primer pair ANG1018/ANG1019. The kanamycin marked *gdpP* deletion was then transduced into a fresh SEJ1 and LAC* strain background yielding strains SEJ1Δ*gdpP*::*kan* (ANG1959) and LAC*Δ*gdpP*::*kan* (ANG1961), respectively.

**Western immunoblotting** Western immunoblotting was performed as described previously . In brief, LTA was extracted from 1 ml cultures and samples normalized based on OD_600_ readings. Samples were boiled for 20 min, centrifuged at 17,000 x *g* for 5 min and 10 μl aliquots separated on 15% SDS-PAGE gels and subsequently transferred to a PVDF membrane. LTA was detected using the monoclonal polyglycerolphosphate-specific LTA antibody (1:4,000; Clone 55 from Hycult Biotechnology) and the HRP-conjugated anti-mouse IgG (1:10,000; Cell Signaling Technologies, USA) and blots were developed by enhanced chemiluminescence.

**Autolysis assay** SEJ1, SEJ1Δ*atl* and the suppressor strains were grown overnight in TSB medium and then backdiluted 1:100 in fresh medium and grown to an OD_600_ of 1. Samples corresponding to an OD_600_ of 1.4 were centrifuged, washed twice in 10 mM sodium phosphate buffer pH 7.0 and twice in ice-cold ddH_2_O before suspension in 10 mM sodium phosphate buffer pH 7.0 containing 0.05% (v/v) Triton X-100. Cells were incubated at 37°C and autolysis monitored by measuring OD_600_ values over a 5 h time period. Experiments were done in triplicate. Representative graphs are shown.

**Zymographic analysis** Overnight cultures were backdiluted 1:100 into 5 ml TSB, with antibiotic where required, and grown to an OD_600_ of 1. The 5 ml culture was centrifuged, the pellet suspended in 40 μl sample buffer and boiled for 20 min. Samples were centrifuged at 17,000 x *g* for 5 min and 20 μl was loaded onto 7.5% SDS-PAGE gels containing 3% (w/v) heat-killed *Micrococcus luteus*. Gels were washed twice in ddH_2_O for 30 min each before overnight incubation in 0.2 M phosphate buffer pH 7.0 at 37°C. Autolytic activity was observed as clear zones against an opaque background. Gels were subsequently stained with 0.5% methylene blue to aid with visualization.

**Minimum Inhibitory Concentrations** Overnight cultures of WT, Δ*gdpP* and suppressor strains as indicated in the text were adjusted to 5 x 10^5^ bacteria/ml in Miller Hinton broth and 100 μl of these suspensions were incubated in 96 well plates with 2-fold dilutions of various antimicrobials at the following starting concentrations: oxacillin 1.024 mg/ml or 1 μg/ml as appropriate, daptomycin 32 μg/ml, lysostaphin 16 μg/ml, vancomycin 32 μg/ml, nisin 50 μg/ml and penicillin G 51.2 μg/ml or 0.2 μg/ml as appropriate. Oxacillin and daptomycin containing wells were supplemented with 2% (w/v) NaCl and 0.23 mM CaCl_2_, respectively. Plates were incubated at 37°C overnight with shaking. MICs were determined as the concentration of the antimicrobial at which growth was inhibited by >75% compared to growth without antimicrobial.

**Phase contrast and fluorescence microscopy** Overnight cultures of SEJ1 and the suppressor strains were diluted 1:100 and grown in TSB medium to an OD_600_ of 1. SEJ1Δ*ltaS* strains were grown overnight in TSB 7.5% NaCl or 40% sucrose, diluted 1:50 into TSB 7.5% NaCl or 40% sucrose and grown to an OD_600_ of 1. To determinate the cell diameter, overnight cultures of SEJ1 SEJ1Δ*gdpP*, 4S5, LAC*, LAC*Δ*gdpP::kan,* LAC*Δ*gdpP::kan* pRMC3 and LAC*Δ*gdpP::kan* pRMC3-*gdpP* were diluted 1:100 in TSB and grown for 3 to 4 h in TSB medium plus antibiotic where appropriate. Cells from 1 ml culture were washed 3 times in PBS pH 7.4 before 150 μl were applied to polylysine (0.1%, w/v) treated coverslips. Bacteria-coated coverslips were incubated with 1 μg/ml BODIPY-vancomycin for 5 min, washed and mounted on glass slides with 5% (w/v) N-propyl gallate. Slides were viewed under a Zeiss Axiovert 200 wild-field microscope using a 100x objective and images were taken and analyzed using Improvision Volocity software. Five images were taken of random fields of view for each strain. Each microscope image was divided into 9 squares and within each square the diameter of all the cells of the following type were measured: single cells (without new division septum in place) or doublet cells that had divided but were attached at a small point. After measuring all the cells of this type in the first square, all cells in the second square were measured and so on until a total of 20 cells was reached (usually after measuring the diameters of all cells within the top 3 out of 9 squares). This was repeated for the 5 pictures, resulting in a total of 100 cells/strain. The experiment was performed in triplicate and therefore the average diameters are based on measuring the diameters of 300 cells.

**Biofilm formation** *S. aureus* strains SEJ1, SEJ1Δ*gdpP* and SEJ1Δ*gdpP::kan* were grown overnight in TSB and backdiluted to an OD_600_ of 0.025 in BHI supplemented with NaCl (4%, w/v). Two hundred μl of these cultures were added to sterile 96-well polystyrene plates (VWR) and incubated at 37°C for 24 h. Wells were washed 3 times with PBS pH 7.4 and dried by inversion for 1 h at room temperature. Adherent bacteria were stained with 100 μl of a 0.5% (w/v) crystal violet solution, the stain was removed and the wells were washed 3 times with PBS pH 7.4. Any adhering stain was solubilized with 100 μl of 5 % (v/v) acetic acid and the A_620_ measured. Three independent experiments were performed with triplicate samples and the average and standard deviations of the three values from one representative experiment are depicted.

**Quantification of c-di-AMP by LC-MS/MS** The chromatographic separation was performed on a Series 200 HPLC system (Perkin Elmer Instruments, Norwalk, CT, USA) equipped with a binary pump system and a 200 μl sample loop. A combination of Supelco Column Saver (2.0 μm filter, Supelco Analytical, Bellafonte, CA, USA), Security Guard Cartridge (C18, 4 x 2 mm) in an Analytical Guard Holder KJO-4282 (Phenomenex, Aschaffenburg, Germany) and an analytical NUCLEODUR C18 Pyramid RP column (50 x 3 mm, 3 μm particle size, Macherey-Nagel, Düren, Germany) temperature controlled by a convenient HPLC column oven (Series 200 Peltier column oven, Perkin Elmer Instruments) at 30°C was used. Eluent A consisted of 10 mM ammonium acetate and 0.1% (v/v) acetic acid in water and eluent B was methanol. The injection volume was 50 μl and the flow rate was 0.4 ml/min throughout the chromatographic run. 100% A was used from 0 to 5 minutes followed by a linear gradient from 100% A to 70% A until 9 minutes. The internal standard cXMP and ^13^C^15^N-c-di-AMP, respectively, and c-di-AMP were eluted during this gradient phase with retention times of 6.1 for cXMP and 9.1 minutes for c-di-AMP and ^13^C^15^N-c-di-AMP, respectively. Re-equilibration of the column was achieved by constantly running 100% A from 9 to 13 minutes.

The analyte detection was performed on an API 3000 triple quadrupole mass spectrometer equipped with an electrospray ionization (ESI) source (Applied Biosystems Inc, Foster City, CA, USA) using selected reaction monitoring (SRM) analysis in positive ionization mode. The following SRM transitions using a dwell time of 40 ms were detected: cXMP: +347/153 (quantifier), +347/136 (identifier); ^13^C^15^N-c-di-AMP: +689/146 (quantifier), +689/345 (identifier) and c-di-AMP: +659/136 (quantifier), +659/330 (identifier) and +659/524 (identifier). The SRM transitions labeled as “quantifier” were used to quantify the compound of interest whereas “identifier” SRM transitions were monitored as confirmatory signals. The quantifier SRM transitions were most intense and were therefore used for quantification. The mass spectrometer parameters were as follows: IS voltage: 5500 V, temperature: 350°C, nebulizer gas: 6 psi, curtain gas: 15 psi. MS/MS was performed using nitrogen as collision gas. The following collision energies were applied: 29 eV (+347/153), 59 eV (+347/136), 61 eV (+689/146), 29 eV (+689/345), 63 eV (+659/136), 29 eV (+659/330), 33 eV (+659/524).

**Supplementary Figures**

**
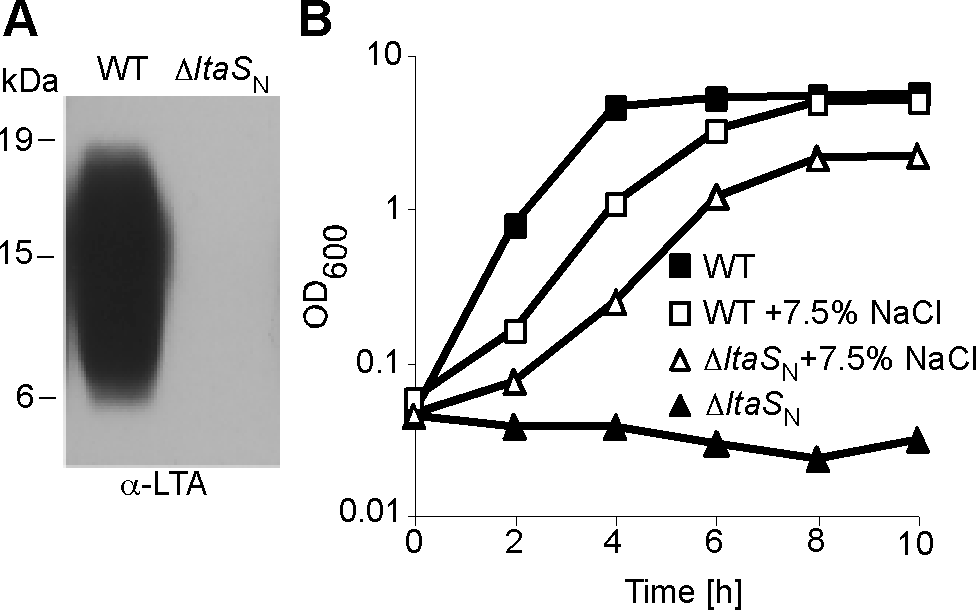
**

**Figure S1.** **Growth and LTA production of *S. aureus* WT versus *ltaS* deletion strains.** (A) LTA detection by western blot. *S. aureus* strains SEJ1 (WT) and SEJ1Δ*ltaS*_N_ (Δ*ltaS*_N_) were grown in TSB 7.5% NaCl medium, cell-associated LTA was extracted and analyzed by western blot using a monoclonal anti-LTA antibody. The positions of protein molecular mass markers (in kDa) are indicated on the left. (B) Bacterial growth curves. Overnight cultures of WT and Δ*ltaS*_N_*,* both grown in TSB 7.5% NaCl, were washed 3 times in either TSB or TSB 7.5% NaCl and diluted to a starting OD_600_ of 0.05 in their respective broth. Growth was monitored over a 10 h period by sampling and determining OD_600_ values every 2 h.


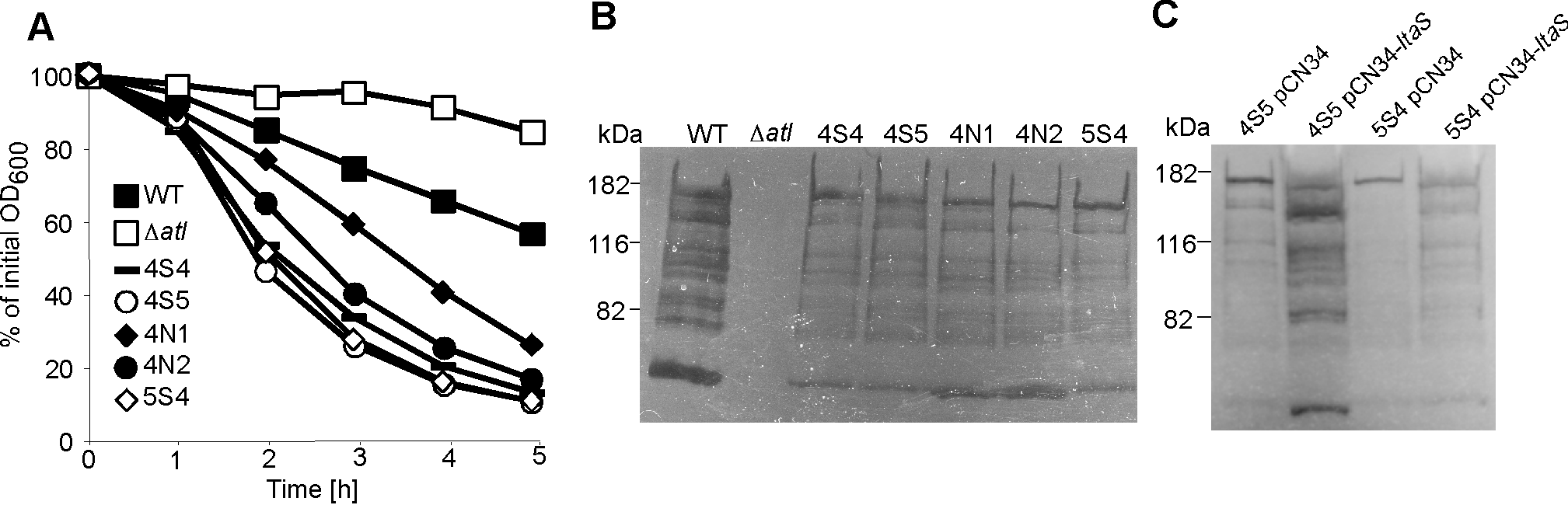


**Figure S2. Phenotypic analysis of LTA-negative *S. aureus* suppressor strains.** (A) Autolysis assay. Triton X-100 induced autolysis assays were performed as described in the supplementary materials and methods section and OD_600_ values determined over a 5 h period and plotted. Zymogram analysis of (B) suppressor strains and (C) suppressor strains 4S5 or 5S4 containing either the empty vector pCN34 or the LtaS complementation vector pCN34-*ltaS*. Equal amounts of cell wall-associated proteins from bacteria in log phase were loaded onto 7.5% SDS-PAGE gels containing heat-killed *M. luteus*. The negative control, Δ*atl*, refers to strain ANG406 which contains a transposon insertion within *atl*, the gene encoding for the major autolysin Atl. Autolytic enzymes are visualized as clear zones against an opaque background. Gels were subsequently stained with 0.5% methylene blue to aid with visualization. The inverse images are shown. Strains used are indicated on the top and the positions of protein molecular mass markers (in kDa) are indicated on the left.

**
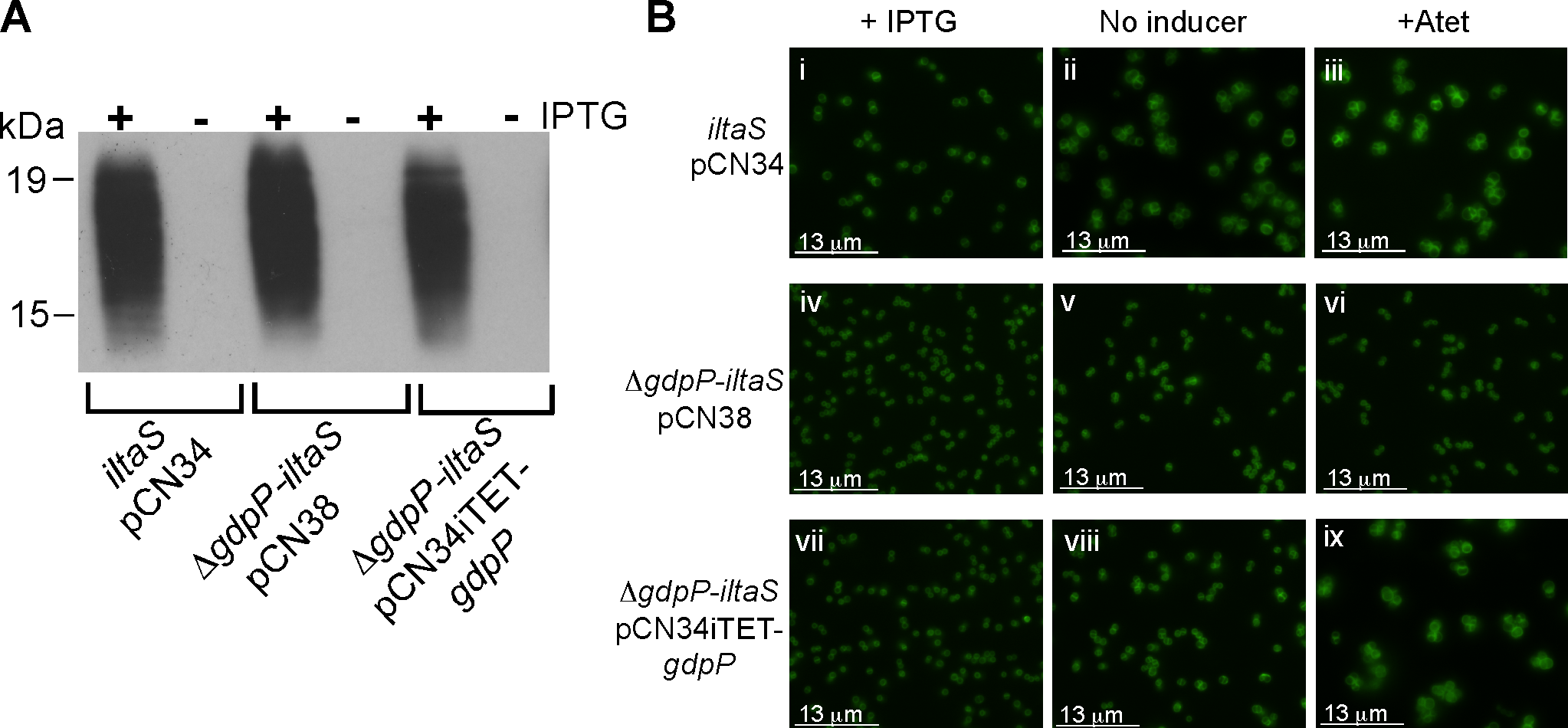
**

**Figure S3. Characterization of RN4220*iltaS*Δ*gdpP* strains.** (A) *S. aureus* strains RN4220*iltaS* pCN34 (*iltaS* pCN34), SEJ1Δ*gdpP*-*iltaS* pCN38 (Δ*gdpP*-*iltaS* pCN38) and SEJ1Δ*gdpP*-*iltaS* pCN34iTET-*gdpP* (Δ*gdpP*-*iltaS* pCN34iTET-*gdpP*) were grown for 4h in TSB ± 1 mM IPTG and LTA analyzed by western blot. (B) Microscopic analysis of strains RN4220*iltaS* pCN34 (*iltaS* pCN34; i - iii), SEJ1Δ*gdpP*-*iltaS* pCN38 (Δ*gdpP*-*iltaS* pCN38; iv - vi) and SEJ1Δ*gdpP*-*iltaS* pCN34iTET-*gdpP* (Δ*gdpP*-*iltaS* pCN34iTET-*gdpP*; vii - ix) was performed as described in Figure 3B but larger fields of view are shown.

**
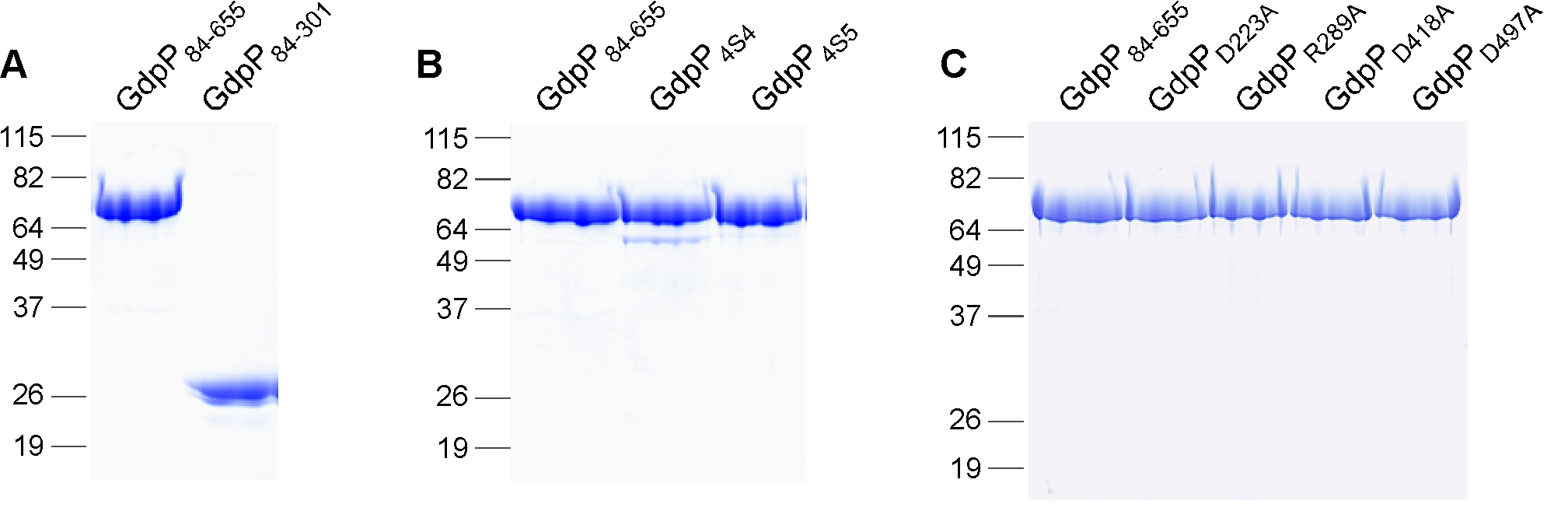
**

**Figure S4. Coomassie stained gels of purified recombinant *S. aureus* rGdpP variants.** The different recombinant GdpP variants were expressed as N-terminal His-tag fusion proteins in *E. coli* and purified by Ni-affinity and size exclusion chromatography as described in the supplementary materials and methods section. Ten μg of (A) GdpP_84-655_ and GdpP_84-301_ (B) GdpP_84-655_, GdpP_4S4_ and GdpP_4S5_ (C) GdpP_84-655_, GdpP_D223A_, GdpP_R289A_, GdpP_D418A_ and GdpP_D497A_ protein variants were run on 10% PAA-gels and proteins visualized by staining with coomassie brilliant blue. Sizes (in kDa) of protein standards run in parallel are indicated on the left of each panel.


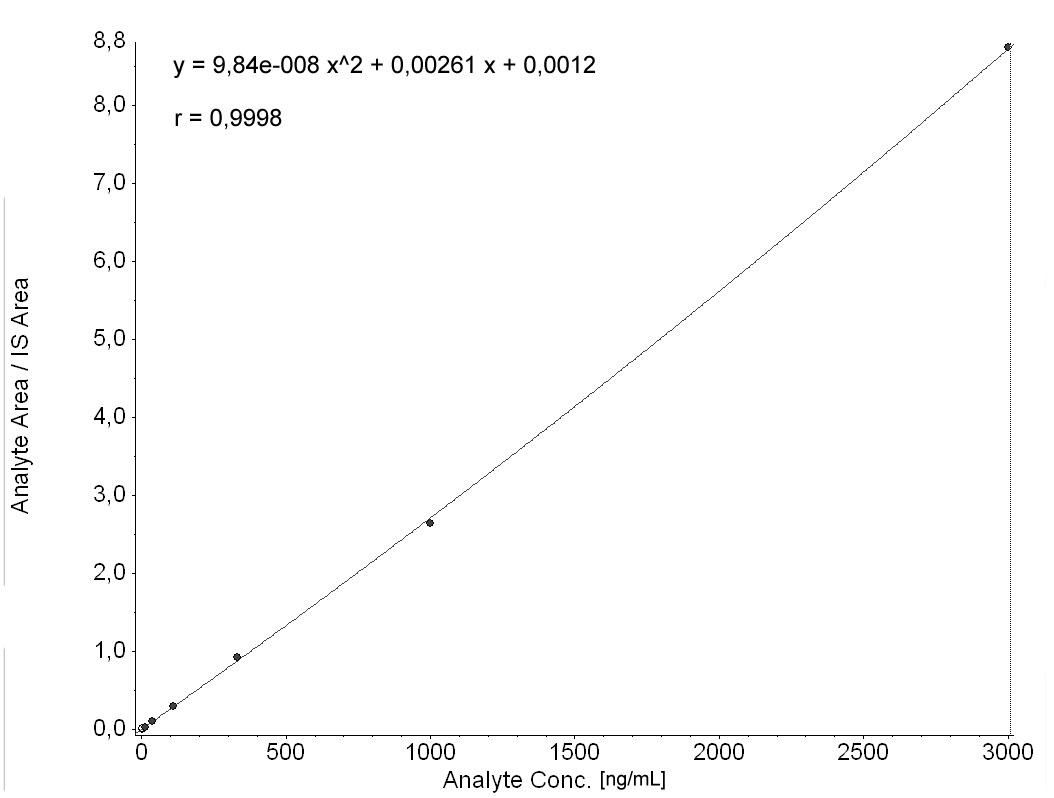


**Figure S5. Calibration curve for the quantification of c-di-AMP by LC-MS/MS.** A calibration curve was established by quantifying the c-di-AMP-specific mass spectrometry signals of solutions containing c-di-AMP calibrators at known concentrations ranging from 1.37 to 3000 ng/ml and containing the isotope-labeled internal standard ^13^C^15^N-c-di-AMP at a fixed concentration of 0.58 μM. The ratios of the c-di-AMP to the isotope-labeled internal standard peak areas (c-di-AMP/^13^C^15^N-c-di-AMP) are plotted against the c-di-AMP concentration in ng/ml. A quadratic regression curve was used to fit the data points and for the determination of c-di-AMP concentrations in *S. aureus* extracts.

**
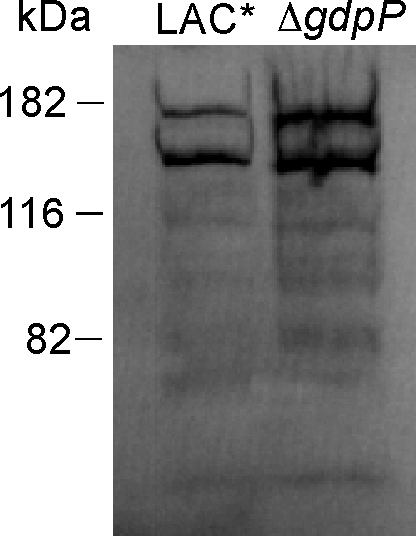
**

**Figure S6. Zymogram analysis of LAC* and LAC*Δ*gdpP::kan* strains.** Equal amounts of cell wall-associated proteins from LAC* and LAC*Δ*gdpP::kan* grown to log phase were loaded onto 7.5% SDS-PAGE gels containing heat-killed *M. luteus*. Autolytic enzymes are visualized as clear zones against an opaque background. The inverse images are shown. Strains used are indicated on the top and the positions of protein molecular mass markers (in kDa) are indicated on the left.


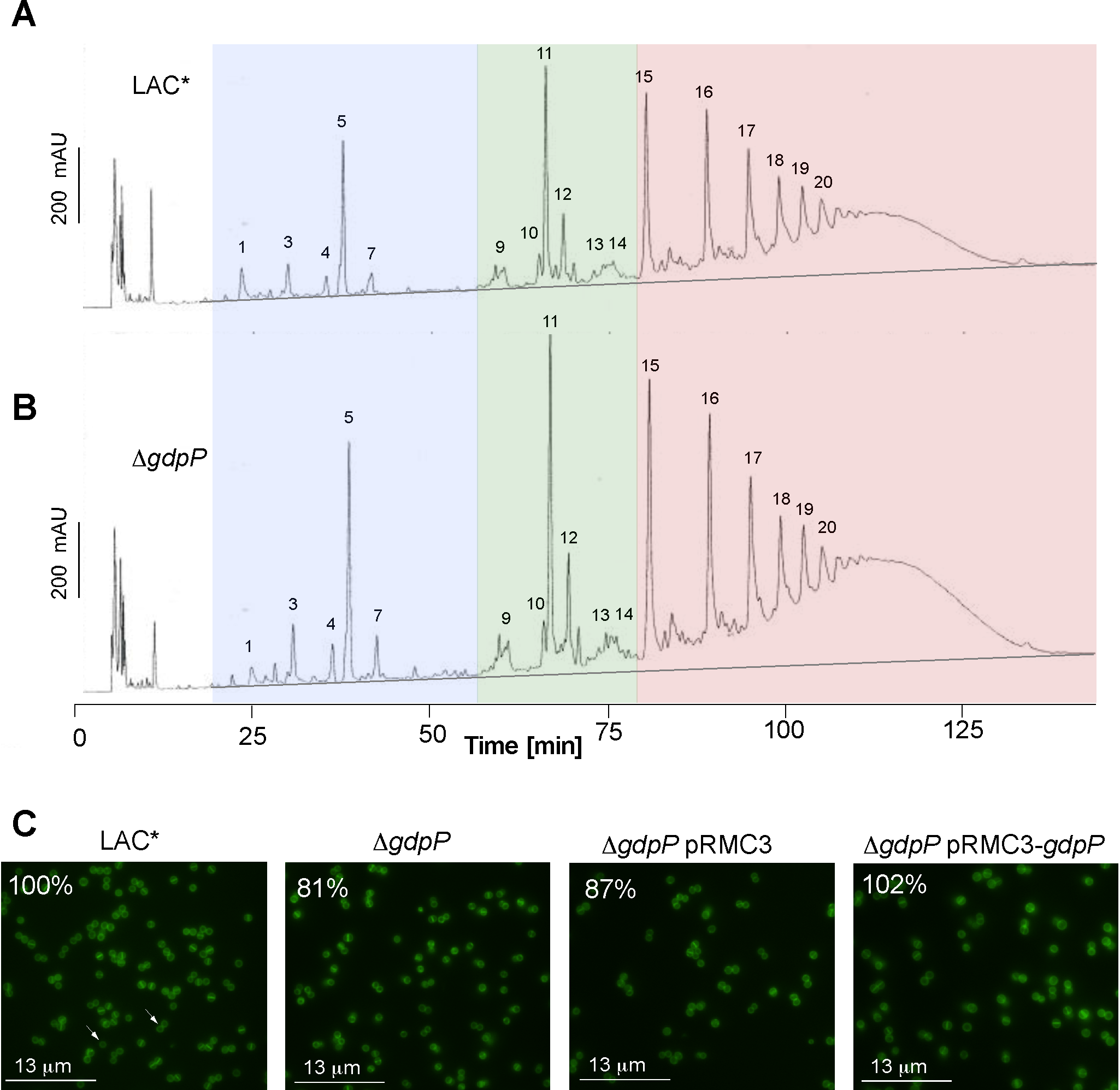


**Figure S7. Muropeptide and microscopic analysis of LAC* and LAC*Δ*gdpP::kan* strains.** HPLC profiles of muropeptides derived from *S. aureus* strains (A) LAC* and (B) LAC*Δ*gdpP::kan*. Peptidoglycan was isolated, digested with mutanolysin and separated by HPLC as described in the material and methods section. Traces were recorded at 205 nm and are shown with monomer muropeptide peaks highlighted in blue, dimer peaks in green and trimer & above peaks in red. Muropeptide peaks are numbered as described in de Jonge *et. al.* . (C) Microscopic analysis of LAC*, LAC*Δ*gdpP::kan,* LAC*Δ*gdpP::kan* pRMC3 and LAC*Δ*gdpP::kan* pRMC3-*gdpP* was performed as described in Figure 8B but larger fields of view are shown.

**
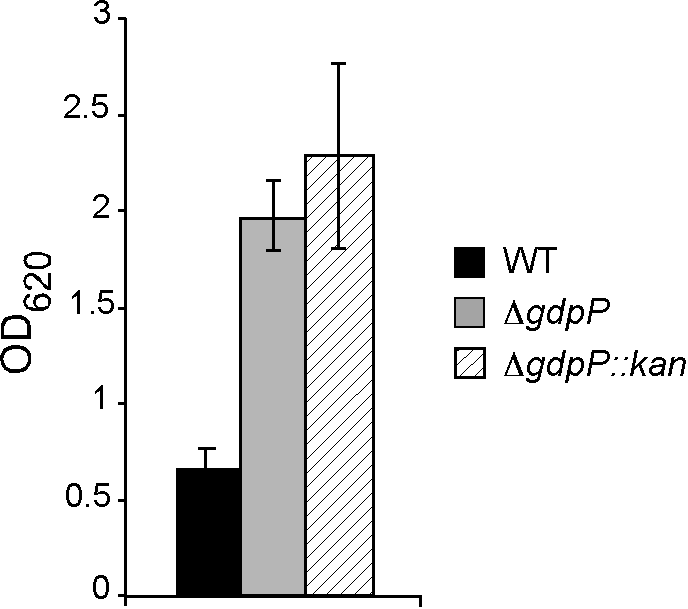
**

**Figure S8.** **Biofilm formation.** The biofilm forming capacity of SEJ1 (WT – black bars), SEJ1Δ*gdpP* (Δ*gdpP* –grey bars) and SEJ1Δ*gdpP::kan* (Δ*gdpP::kan* – striped bars) were determined by growing the different cultures for 24 h in 96-well plates in BHI broth containing 4% NaCl. Non-adherent bacteria were removed by washing wells with PBS buffer and remaining adherent bacteria were stained with crystal violet. Unbound crystal violet dye was removed by washing wells again with PBS buffer and the remaining stain bound to adherent bacteria was dissolved in 100 μl of 5% acetic acid and A_620_ readings determined and plotted. Three independent experiments were performed with triplicate samples and the average and standard deviations of the three values from one representative experiment are shown.

**Supplementary Tables**

**Table S1. MICs for different antibiotics given in μg/ml**

|  | **SEJ1** | **4S4** | **4S5** | **4N1** | **4N2** | **5S4** | **Mode of action** |
| --- | --- | --- | --- | --- | --- | --- | --- |
| **Lysostaphin** | 0.5 | 0.125 | 0.25 | 0.25 | 0.25 | 0.25 | Peptidoglycan – cleaves pentaglycine crossbridges |
| **Oxacillin** | 0.125 | 0.062 | 0.062 | 0.062 | 0.062 | 0.062 | Peptidoglycan – inhibits PBPs and peptidoglycan crosslinking |
| **Penicillin G** | 0.05 | 0.0125 | 0.025 | 0.025 | 0.025 | 0.025 | Peptidoglycan – inhibits PBPs and peptidoglycan crosslinking |
| **Vancomycin** | 4 | 1 | 2 | 2 | 1 | 1 | Peptidoglycan – binds to D-Ala D-Ala and prevents peptidoglycan crosslinking |
| **Nisin** | 12.5 | 3.125 | 3.125 | 3.125 | 3.125 | 3.125 | Antimicrobial peptide |
| **Daptomycin** | 4 | 2 | 2 | 2 | 2 | 1 | Membrane depolarization/disruption |

MICs are defined as antibiotic concentration that leads to >75 % growth inhibition as compared to growth without antibiotic

**Table S2. *gdpP* mutations in LAC*Δ*ltaS* suppressor strains**

| **Strain** | **Nucleotide substitution** | **Amino acid substitution** |
| --- | --- | --- |
| **UN1** | large rearrangement of 5' end based on PCR analysis | Large rearrangement |
| **UN2** | None | None |
| **UN3** | A insertion after base 19,714 | Y457stop |
| **UN4** | GA 18,788/18,789 CG and A insertion at 18,790 | 150stop |
| **US1** | None | None |
| **US2** | G 19,617 T | V425F |
| **US3** | A insertion after 19,035 | 236stop |
| **US4** | None | None |

Numbers indicate location of mutations in the USA300_TCH1516 genome

**Table S3. Bacterial strains used in this study**

| **Strain** | **Relevant features** | **Reference** |
| --- | --- | --- |
|  | ***Escherichia coli* strains** |  |
| XL1-Blue | Cloning strain, TetR – ANG127 | Stratagene |
| DH5α | Cloning strain – ANG397 |  |
| BL21(DE3) | Strain used for protein expression – ANG191 | Novagen |
| ANG126 | pTS1 in DH5α: AmpR |  |
| ANG201 | pCN34 in *E. coli*; source for Kan (*aphA*-3) marker: AmpR |  |
| ANG203 | pCN49 in *E. coli*; source of transcription terminators (TT): AmpR |  |
| ANG474 | pMUTIN-HA in *E. coli*; source for Erm (*ermAM*) marker: AmpR | Bacillus genetic stock center |
| ANG498 | pMUTIN–HA–*ltaS* in XL1–Blue: AmpR |  |
| ANG503 | pCL55-*ltaS* in XL1-Blue: AmpR |  |
| ANG1222 | pCN34-*ltaS* in XL1-Blue: AmpR | This study |
| ANG1429 | pKOR1 in *E. coli* strain DB3.1: AmpR |  |
| ANG1550 | pRMC2 in *E. coli*; *S. aureus*/*E. coli* shuttle vector. Source of the *tetR* repressor gene and *xyl/tetO* promoter: AmpR |  |
| ANG1578 | pKOR1-Δ*ltaS* in DH5α: AmpR | This study |
| ANG1632 | pCN34iTET in XL1-Blue; *E. coli* / *S. aureus* shuttle vector with tetracycline inducible promoter from pRMC2: AmpR | This study |
| ANG1676 | pCN38 in *E. coli*; source for Gram-positive Cam (*cat194*) marker: AmpR |  |
| ANG1764 | pCN34iTET-*gdpP* in XL1-Blue: AmpR | This study |
| ANG1766 | pKOR1-Δ*ltaS*::*erm* in DH5α: AmpR | This study |
| ANG1791 | pTS1-Δ*gdpP* in XL1-Blue: AmpR | This study |
| ANG1834 | pCN34iTET-*gdpP*_4S4_ in XL1-Blue: AmpR | This study |
| ANG1835 | pCN34iTET-*gdpP*_4S5_ in XL1-Blue: AmpR | This study |
| ANG1836 | pCN34iTET-*gdpP*_4N2_ in XL1-Blue: AmpR | This study |
| ANG1854 | pET28b-*gdpP*_84-655_ in XL1-Blue: KanR | This study |
| ANG1857 | pET28b-*gdpP*_84-301_ in XL1-Blue: KanR | This study |
| ANG1861 | pET28b-*gdpP*_84-655_ in BL21(DE3): KanR | This study |
| ANG1864 | pET28b-*gdpP*_84-301_ in BL21(DE3): KanR | This study |
| ANG1867 | pET28b in BL21(DE3): KanR | Novagen |
| ANG1878 | pCN34iTET-*gdpP*_D223A_ in XL1-Blue: AmpR | This study |
| ANG1879 | pCN34iTET-*gdpP*_R289A_ in XL1-Blue: AmpR | This study |
| ANG1880 | pCN34iTET-*gdpP*_D418A_ in XL1-Blue: AmpR | This study |
| ANG1881 | pCN34iTET-*gdpP*_D497A_ in XL1-Blue: AmpR | This study |
| ANG1945 | pET28b-*gdpP*_D223A_ in XL1-Blue: KanR | This study |
| ANG1946 | pET28b-*gdpP*_R289A_ in XL1-Blue: KanR | This study |
| ANG1947 | pET28b-*gdpP*_D418A_ in XL1-Blue: KanR | This study |
| ANG1948 | pET28b-*gdpP*_D497A_ in XL1-Blue: KanR | This study |
| ANG1949 | pET28b-*gdpP*_D223A_ in BL21(DE3): KanR | This study |
| ANG1950 | pET28b-*gdpP*_R289A_ in BL21(DE3): KanR | This study |
| ANG1951 | pET28b-*gdpP*_D418A_ in BL21(DE3): KanR | This study |
| ANG1952 | pET28b-*gdpP*_D497A_ in BL21(DE3): KanR | This study |
| ANG1954 | pKOR1-Δ*gdpP*::*kan* in DH5α: AmpR | This study |
| ANG1965 | pET28b-*disA* in XL1-Blue: KanR | This study |
| ANG1966 | pET28b-*dacA* in XL1-Blue: KanR | This study |
| ANG1970 | pET28b-*disA* in BL21(DE3): KanR | This study |
| ANG1971 | pET28b-*dacA* in BL21(DE3): KanR | This study |
| ANG2050 | pET28b-*gdpP*_4S4_ in XL1-Blue: KanR | This study |
| ANG2051 | pET28b-*gdpP*_4S5_ in XL1-Blue: KanR | This study |
| ANG2052 | pET28b-*gdpP*_4S4_ in BL21(DE3): KanR | This study |
| ANG2053 | pET28b-*gdpP*_4S5_ in BL21(DE3): KanR | This study |
| ANG2054 | pCN38iTET in XL1-Blue; pCN34iTET with Cam in place of Kan marker; AmpR | This study |
| ANG2055 | pCN38iTET-TT in XL1-Blue: AmpR | This study |
| ANG2056 | pRMC3 in XL1-Blue: AmpR | This study |
| ANG2058 | pRMC3-*gdpP* in XL1-Blue:AmpR | This study |
|  |  |  |
|  | ***Staphylococcus aureus* strains** |  |
| RN4220 | Restriction deficient derivative of 8325-4 |  |
| SEJ1 | RN4220Δ*spa;* protein A negative derivative of RN4220; ANG314 |  |
| ANG406 | SEJ1Δ*atl* | Lab strain collection |
| ANG499 | RN4220*iltaS*: ErmR, IPTG |  |
| ANG1130 | SEJ1*-iltaS* pCN34: ErmR, KanR, IPTG |  |
| ANG1433 | SEJ1Δ*ltaS* pCN34-*ltaS*: KanR | This study |
| ANG1480 | SEJ1Δ*ltaS*_N_ pCN38; Isolated on 7.5% NaCl: CamR | This study |
| ANG1481 | SEJ1Δ*ltaS*_S_ pCN38; Isolated on 40% Sucrose: CamR | This study |
| LAC* | LAC*: Erm sensitive CA-MRSA LAC strain (AH1263) |  |
| ANG1677 | *Micrococcus luteus* NCTC2665 |  |
| ANG1765 | SEJ1 pCN34iTET-*gdpP*: KanR, ± Atet | This study |
| ANG1774 | SEJ1 pCN34-*ltaS*: KanR | This study |
| ANG1775 | SEJ1Δ*ltaS*::*erm* pCN34-*ltaS*: KanR and ErmR | This study |
| ANG1780 | LAC* pCN34-*ltaS:* KanR | This study |
| ANG1782 | LAC*Δ*ltaS*::*erm* pCN34-*ltaS*: KanR and ErmR | This study |
| ANG1785 | 4S4: SEJ1Δ*ltaS*_S_ suppressor strain | This study |
| ANG1786 | 4S5: SEJ1Δ*ltaS*_S_ suppressor strain | This study |
| ANG1787 | 4N1: SEJ1Δ*ltaS*_N_ suppressor strain | This study |
| ANG1788 | 4N2: SEJ1Δ*ltaS*_N_ suppressor strain | This study |
| ANG1789 | 5S4: SEJ1Δ*ltaS*_S_ suppressor strain | This study |
| ANG1792 | 4S5 pCN34iTET-*gdpP*: KanR, Atet | This study |
| ANG1795 | SEJ1Δ*gdpP* pCN38: CamR | This study |
| ANG1798 | 4S5 pCN34iTET: KanR, Atet | This study |
| ANG1837 | 4S5 pCN34iTET-*gdpP*_4S4_: KanR, ± Atet | This study |
| ANG1838 | 4S5 pCN34iTET-*gdpP*_4S5_: KanR, ± Atet | This study |
| ANG1839 | 4S5 pCN34iTET-*gdpP*_4N2_: KanR, ±Atet | This study |
| ANG1849 | SEJ1Δ*gdpP-iltaS* pCN38: ErmR, CamR, IPTG | This study |
| ANG1883 | 4S5 pCN34iTET-*gdpP*_D223A_: KanR, ± Atet | This study |
| ANG1884 | 4S5 pCN34iTET-*gdpP*_R289A_: KanR, ± Atet | This study |
| ANG1885 | 4S5 pCN34iTET-*gdpP*_D418A_: KanR, ± Atet | This study |
| ANG1886 | 4S5 pCN34iTET-*gdpP*_D497A_: KanR, ± Atet | This study |
| ANG1958 | SEJ1Δ*gdpP*::*kan*: KanR | This study |
| ANG1959 | SEJ1Δ*gdpP*::*kan*: clean strain: KanR | This study |
| ANG1961 | LAC*Δ*gdpP*::*kan*: KanR | This study |
| ANG2113 | SEJ1Δ*gdpP-iltaS* pCN34iTET-*gdpP*: ErmR, KanR, IPTG, ± Atet | This study |
| ANG2134 | LAC*Δ*ltaS*_N_::*erm* pCN38; Isolated on 7.5% NaCl: CamR and ErmR | This study |
| ANG2135 | LAC*Δ*ltaS*_S_::*erm* pCN38; Isolated on 40% Sucrose: CamR and ErmR | This study |
| ANG2136 | UN1: LAC*Δ*ltaS*_N_::*erm* suppressor | This study |
| ANG2137 | UN2: LAC*Δ*ltaS*_N_::*erm* suppressor | This study |
| ANG2138 | UN3: LAC*Δ*ltaS*_N_::*erm* suppressor | This study |
| ANG2139 | UN4: LAC*Δ*ltaS*_N_::*erm* suppressor | This study |
| ANG2140 | US1: LAC*Δ*ltaS*_S_::*erm* suppressor | This study |
| ANG2141 | US2: LAC*Δ*ltaS*_S_::*erm* suppressor | This study |
| ANG2142 | US3: LAC*Δ*ltaS*_S_::*erm* suppressor | This study |
| ANG2143 | US4: LAC*Δ*ltaS*_S_::*erm* suppressor | This study |
| ANG2232 | 4S5 pCN34: KanR | This study |
| ANG2233 | 4S5 pCN34-*ltaS*: KanR | This study |
| ANG2234 | 5S4 pCN34: KanR | This study |
| ANG2235 | 5S4 pCN34-*ltaS*: KanR | This study |
| ANG2236 | LAC*Δ*gdpP*::*kan* pRMC3: KanR, CamR | This study |
| ANG2237 | LAC*Δ*gdpP*::*kan* pRMC3-*gdpP*: KanR, CamR | This study |
|  |  |  |
|  | ***Bacillus subtilis* strains** |  |
| ANG1691 | *Bacillus subtilis* 168 – Transformable laboratory strain, trpC2 |  |
|  |  |  |

Antibiotics were used at the following concentrations: for *E. coli* cultures: Ampicillin (AmpR) 100 μg/ml; Kanamycin (KanR) 30 μg/ml; for *S. aureus* cultures: Erythromycin (ErmR) 10 μg/ml; Kanamycin (KanR) 90 μg/ml; Chloramphenicol (CamR) 5 to 10 μg/ml, IPTG at 1 mM and Anhydrotetracycline (Atet) 100 or 200 ng/ml.

**Table S4. Primers used in this study**

| **Number** | **Name** | **Sequence** |
| --- | --- | --- |
| ANG86 | F-BamHI-promoter ltaS | CGGGATCCGGAATAGAATATAGAATGCAATTAGAAATG |
| ANG87 | R-SalI-LtaS | ACGCGTCGACCCGAGTTCGTGTTTAAATATTATTTTTTAG |
| ANG241 | F-AttB1-upLtaS_pKOR1 | GTACAAAAAAGCAGGCTGGGAGGCGGATTTGAATTAGATGCATGGTTAGG |
| ANG572 | R-AttB2-downLtaS_pKOR1 | GGGGACCACTTTGTACAAGAAAGCTGGGTGCTCGTTGCTTTTGTTGTTGC |
| ANG669 | F-KpnI-downLtaS_pKOR1 | GGGGGTACCGATAAAATATCAATCCGGGTT |
| ANG671 | R-KpnI-upLtaS_pKOR1 | GGGGGTACCATCTTGCGAAAGTACTTG |
| ANG849 | F5'downLtaS-3'ErmAM_pKOR1 | GGGAGGAAATAAGATAAAATATCAATCCGGGTTTTT |
| ANG850 | F5'ErmAM-3'upLtaS_pKOR1 | CTTTCGCAAGATACTCTTCCTTTTTCAATATTATTG |
| ANG851 | R3'ErmAM-5'downLtaS_pKOR1 | TGATATTTTATCTTATTTCCTCCCGTTAAATAATAG |
| ANG852 | R3'upLtaS-5'ErmAM_pKOR1 | AAAAGGAAGAGTATCTTGCGAAAGTACTTGAATAAA |
| ANG908 | F-XmaI-iTET | TCCCCCGGGcgGAATTCGAGCTCAGATCTGTTAACGGTACCatc |
| ANG921 | F-KpnI-GdpP | tttGGTACCctaaaaagtgaatagag |
| ANG922 | R-EcoRI-GdpP | cccGAATTCcttTCATGCATCTTCACTC |
| ANG946 | F-AttB1 | GGGGACAAGTTTGTACAAAAAAGCAGGCTTC |
| ANG947 | R-AttB2 | GGGGACCACTTTGTACAAGAAAGCTGGGTC |
| ANG948 | R-NarI-iTET | CCTTGGCGCCTTAAGACCCACTTTCACATTTAAGTTG |
| ANG990 | F-AttB1-upGdpP_pKOR1 | ACAAAAAAGCAGGCTTCttcaattaaatgaaatagaagcatacaatc |
| ANG991 | R-AttB2-downGdpP_pKOR1 | ACAAGAAAGCTGGGTCCTCTTCAGCTGTTTCATACACTTGTCCTAA |
| ANG992 | F5'00017-3'00014_pTS1 | tgaatagaggtgAAGTAGGAGTGAAGATGCATGAAA |
| ANG993 | R3'00014-5'00017_pTS1 | TCACTCCTACTTcacctctattcactttttagaatt |
| ANG1054 | F-KpnI-5' 00014_pTS1 | gagggtaccttcaattaaatgaaatagaagcatacaatc |
| ANG1067 | R-BamHI-3'00018_pTS1 | cccGGATCCCTCTTCAGCTGTTTCATACACTTGTCCTAA |
| ANG1132 | F-NheI-84AA-GdpP | CTAGCTAGCCCAATTGGTATCATTGTTTTAGATGAAAATG |
| ANG1134 | R-EcoRI-301AA-STOP-GdpP | CGGAATTCtcaACCATTAATACTTTTAATTGCAACTTGG |
| ANG1156 | F-GdpP-D223A | TTTCAAAAGATACAGTTCCGCTCAATTCGTAGCCTATTTA |
| ANG1157 | R-GdpP-D223A | TAAATAGGCTACGAATTGAGCGGAACTGTATCTTTTGAAA |
| ANG1158 | F-GdpP-R289A | CCTAGACTTAGCATTAGGAGCCGGTGGCGACCAAGTTGCAA |
| ANG1159 | R-GdpP-R289A | TTGCAACTTGGTCGCCACCGGCTCCTAATGCTAAGTCTAGG |
| ANG1160 | F-GdpP-D418A | GACAACCGTAGTGATTGTTGCTACGCATAAACCGGAACTG |
| ANG1161 | R-GdpP-D418A | CAGTTCCGGTTTATGCGTAGCAACAATCACTACGGTTGTC |
| ANG1162 | F-GdpP-D497A | GTATGCAGGTATTATTGTAGCTACAAGAAACTTTACATTAC |
| ANG1163 | R-GdpP-D497A | GTAATGTAAAGTTTCTTGTAGCTACAATAATACCTGCATAC |
| ANG1166 | R3'00014-5'Kan_pKOR1 | CATTTTAGCCATTATTCCACCTCTATTCACTTTTTA |
| ANG1167 | F5'Kan-3'00014_pKOR1 | AGAGGTGGAATAATGGCTAAAATGAGAATATCACCG |
| ANG1168 | R3'Kan-5'00017_pKOR1 | CTATCGCCTCTTCTAAAACAATTCATCCAGTAAAAT |
| ANG1169 | F5'00017-3'Kan_pKOR1 | GAATTGTTTTAGAAGAGGCGATAGCACAATTACAAC |
| ANG1205 | F-NdeI-Bs-DisA | TAATAACCATATGGAAAAAGAGAAAAAAGGGG |
| ANG1206 | R-EcoRI-Bs-DisA | CGGAATTCTCACAGTTGTCTGTCTAAATAATGCTTC |
| ANG1209 | F-NcoI-DacA | CATGCCATGGATTTTTCCAACTTTTTTCAAAACC |
| ANG1211 | R-EcoRI-DacA | CGGAATTCggTTTCACACCTTTCTTTTGAAAGCGTGTG |
| ANG1219 | F-XmaI-TT | TTTCCCGGGTATTCTAAATGCATAATAAATACTG |
| ANG1220 | R-SphI-TT | CCCGCATGCTGTCACTTTGCTTGATATATGAG |
| ANG1221 | F-NarI-TT | TTTGGCGCCTATTCTAAATGCATAATAAATACTG |
| ANG1222 | R-NarI-TT | TTTGGCGCCTGTCACTTTGCTTGATATATGAG |
|  |  |  |
|  | Primers for verifying deletions |  |
| ANG247 | FLtaS-check | GATAAAGGCGATGTTTTAAAGTTTAGAGAAAC |
| ANG248 | R-LtaS-check | GATTAATTCTAGCTTGTTGCTTTGTAGTACGAGC |
| ANG1018 | FGdpP-check | CATCGTCATTTCTTCGTGCC |
| ANG1019 | RGdpP-check | CAACTTTTTGTGCAATATTAAGTG |
|  |  |  |

Restriction sites in primer sequences are underlined

**References**

1. Wörmann ME, Corrigan RM, Simpson PJ, Matthews SJ, Gründling A (2011) Enzymatic activities and functional interdependencies of *Bacillus subtilis* lipoteichoic acid synthesis enzymes. Mol Microbiol 79: 566-5683.

2. Charpentier E, Anton AI, Barry P, Alfonso B, Fang Y, et al. (2004) Novel cassette-based shuttle vector system for gram-positive bacteria. Appl Environ Microbiol 70: 6076-6085.

3. Corrigan RM, Foster TJ (2009) An improved tetracycline-inducible expression vector for *Staphylococcus aureus*. Plasmid 61: 126-129.

4. Oku Y, Kurokawa K, Matsuo M, Yamada S, Lee BL, et al. (2009) Pleiotropic roles of polyglycerolphosphate synthase of lipoteichoic acid in growth of *Staphylococcus aureus* cells. J Bacteriol 191: 141-151.

5. O'Connell C, Pattee PA, Foster TJ (1993) Sequence and mapping of the *aroA* gene of *Staphylococcus aureus* 8325-4. J Gen Microbiol 139: 1449-1460.

6. Bae T, Schneewind O (2006) Allelic replacement in *Staphylococcus aureus* with inducible counter-selection. Plasmid 55: 58-63.

7. Gründling A, Schneewind O (2007) Genes Required for Glycolipid Synthesis and Lipoteichoic Acid Anchoring in *Staphylococcus aureus*. J Bacteriol 189: 2521-2530.

8. de Jonge BL, Chang YS, Gage D, Tomasz A (1992) Peptidoglycan composition of a highly methicillin-resistant *Staphylococcus aureus* strain. The role of penicillin binding protein 2A. J Biol Chem 267: 11248-11254.

9. Hanahan D (1983) Studies on transformation of *Escherichia coli* with plasmids. J Mol Biol 166: 557-580.

10. Gründling A, Schneewind O (2007) Synthesis of glycerol phosphate lipoteichoic acid in *Staphylococcus aureus*. Proc Natl Acad Sci U S A 104: 8478-8483.

11. Kreiswirth BN, Lofdahl S, Betley MJ, O'Reilly M, Schlievert PM, et al. (1983) The toxic shock syndrome exotoxin structural gene is not detectably transmitted by a prophage. Nature 305: 709-712.

12. Boles BR, Thoendel M, Roth AJ, Horswill AR (2010) Identification of genes involved in polysaccharide-independent *Staphylococcus aureus* biofilm formation. PLoS One 5: e10146.

13. Fleming A (1929) Arris and Gale lecture on lysozyme, bacteriolytic ferment found normally in tissues and secretions. Lancet 213: 217-270.

14. Burkholder PRaG, N. H (1947) Induced biochemical mutations in *Bacillus subtilis.* Am J Bot 33: 345–348.
